# A systematic review and meta-analysis of diagnostic test accuracy studies of self-report screening instruments for common mental disorders in Arabic-speaking adults

**DOI:** 10.1017/gmh.2021.39

**Published:** 2021-11-23

**Authors:** Anne M. de Graaff, Pim Cuijpers, Mariska Leeflang, Irene Sferra, Jana R. Uppendahl, Ralph de Vries, Marit Sijbrandij

**Affiliations:** 1 Department of Clinical, Neuro- and Developmental Psychology, WHO Collaborating Center for Research and Dissemination of Psychological Interventions, Amsterdam Public Health Institute, Vrije Universiteit Amsterdam, Amsterdam, The Netherlands; 2 Department of Epidemiology and Data Science, Amsterdam University Medical Centers, Amsterdam Public Health, University of Amsterdam, Amsterdam, The Netherlands; 3 Department of Human Neurosciences, Sapienza University of Rome, Rome, Italy; 4 Medical Library, Vrije Universiteit Amsterdam, Amsterdam, The Netherlands

**Keywords:** Anxiety disorders, depressive disorder, self-report, sensitivity and specificity, stress disorders, post-traumatic

## Abstract

**Background:**

Self-report screening instruments are frequently used as scalable methods to detect common mental disorders (CMDs), but their validity across cultural and linguistic groups is unclear. We summarized the diagnostic accuracy of brief questionnaires on symptoms of depression, anxiety and posttraumatic stress disorder (PTSD) among Arabic-speaking adults.

**Methods:**

Five databases were searched from inception to 22 January 2021 (PROSPERO: CRD42018070645). Studies were included when diagnostic accuracy of brief (maximally 25 items) psychological questionnaires was assessed in Arabic-speaking populations and the reference standard was a clinical interview. Data on sensitivity/specificity, area under the curve, and data to generate 2 × 2 tables at various thresholds were extracted. Meta-analysis was performed using the diagmeta package in R. Quality of studies was assessed with QUADAS-2.

**Results:**

Thirty-two studies (*N*_participants_ = 4042) reporting on 17 questionnaires with 5–25 items targeting depression/anxiety (*n* = 14), general distress (*n* = 2), and PTSD (*n* = 1) were included. Seventeen studies (53%) scored high risk on at least two QUADAS-2 domains. The meta-analysis identified an optimal threshold of 11 (sensitivity 76.9%, specificity 85.1%) for the Edinburgh Postnatal Depression Scale (EPDS) (*n*_studies_ = 7, *n*_participants_ = 711), 7 (sensitivity 81.9%, specificity 87.6%) for the Hospital Anxiety and Depression Scale (HADS) anxiety subscale and 6 (sensitivity 73.0%, specificity 88.6%) for the depression subscale (*n*_studies_ = 4, *n*_participants_ = 492), and 8 (sensitivity 86.0%, specificity 83.9%) for the Self-Reporting Questionnaire (SRQ-20) (*n*_studies_ = 4, *n*_participants_ = 459).

**Conclusion:**

We present optimal thresholds to screen for perinatal depression with the EPDS, anxiety/depression with the HADS, and CMDs with the SRQ-20. More research on Arabic-language questionnaires, especially those targeting PTSD, is needed.

## Introduction

Common mental disorders (CMDs) such as depression, anxiety, and posttraumatic stress disorder (PTSD) affect millions of people globally. A meta-analysis across 39 countries indicated a lifetime prevalence of 29.2%, although this estimate varies across subgroups (Demyttenaere *et al*., 2004; Steel *et al*., 2014). Particularly high prevalence rates have been estimated for specific populations, such as refugees and asylum seekers (Steel *et al*., 2009; Charlson *et al*., 2019). Some disorders may be more prevalent because of specific circumstances or group characteristics, however these differences could also reflect the performance of questionnaires across cultures (Gureje and Stein, 2012).

There is a large variety of brief, self-report screening instruments for symptoms of CMDs, such as the Hopkins Symptoms Checklist (HSCL), the Hospital Anxiety and Depression Scale (HADS), and the PTSD Checklist (PCL). Brief instruments can be useful for routine screening in primary and stepped care (Kagee *et al*., 2013; Olin *et al*., 2017), especially where the application of time-consuming, clinician-administered structured interviews is not feasible, such as in low-resource settings (Kohrt *et al*., 2011). Furthermore, the ease of administration of most self-report measures makes them attractive for use in research (Kagee *et al*., 2013). However, these instruments are usually developed and evaluated in specific (Western, Anglo-Saxon) settings (Saxena *et al*., 2006; Ali *et al*., 2016), while psychometric properties may vary across settings, cultures, and languages. For example, in a study on the validity of the HSCL-25 in Lebanon, the optimal cut-off score for anxiety and depression was found to be higher (2.00–2.10) than the widely accepted threshold of 1.75 (Mahfoud *et al*., 2013). This example illustrates the importance of cross-cultural validation of screening tools. The use of thresholds determined in other populations may lead to misclassification and misinterpretation (Steel *et al*., 2009). However, literature on the psychometric properties of screening instruments in cultural contexts outside those for which they were developed is limited (Mutumba *et al*., 2014; Carroll *et al*., 2020; Donnelly and Leavey, 2021).

The ability of a questionnaire (‘index test’) to identify individuals with a CMD compared to individuals without a disorder is called *diagnostic accuracy* (Leeflang *et al*., 2013). Diagnostic accuracy is determined by comparing the outcomes of the index test with the outcomes of a reference standard in the same research subjects. The reference standard is regarded as the best available method to establish the presence or absence of the target condition (Rutjes, 2017). A (semi-structured) clinical interview is the standard for diagnosing mental disorders in clinical practice and mental health research (De Joode *et al*., 2019).

Previous systematic reviews on the validity of screening instruments have focused on a specific instrument (e.g. Edinburgh Postnatal Depression Scale; EPDS) (Gibson *et al*., 2009), outcome (e.g. depression) (Chorwe-Sungani and Chipps, 2017), or income group (e.g. low- and middle-income countries; LAMIC) (Ali *et al*., 2016), but to our knowledge, no systematic review on test performance of brief screening instruments for CMDs in Arabic-speaking populations has been published. Despite the fact that Arabic is one of the most spoken languages in the world, with over 30 dialects and 274 million people that speak Arabic, research on Arabic-language questionnaires is limited (Easton *et al*., 2017; Karnouk *et al*., 2021). Furthermore, last decades have known a steep increase in the number of Arabic-speaking refugees into other parts of the world, such as the Horn of Africa and Europe (UNHCR, 2019, 2021). Psychometrically sound and brief case-finding instruments are vital to scale-up mental health services for an adequate response to the mental health needs of Arabic-speaking refugees worldwide (Jefee-Bahloul *et al*., 2016).

In this systematic review and meta-analysis, we provide an overview of the diagnostic accuracy of Arabic-language psychological distress screening instruments, based on all available evidence in Arabic-speaking adult populations.

## Methods

This review was pre-registered in the International Prospective Register of Systematic Reviews (PROSPERO ID: CRD42018070645). We followed the Preferred Reporting Items for Systematic reviews and Meta-Analyses (PRISMA-DTA) checklist (McInnes *et al*., 2018); see online Supplementary Appendix 1.

### Search strategy

We systematically searched EBSCO/APA PsycINFO, PubMed, Embase.com, Cochrane Library, and Scopus from inception until 22 January 2021, without language restrictions. The search was carried out by a medical information specialist. The following terms were used (including synonyms and closely related words) as index terms or free-text words: ‘Sensitivity and Specificity’, ‘Reference Standards’, ‘Diagnostic Self Evaluation’, ‘Common Mental Disorders’, and ‘Arabic speaking populations’. The full search strategy is attached as online Supplementary Appendix 2. We restricted the search to articles, proceeding papers, conference papers, and electronic collections. We also identified studies by screening literature lists of included studies (Prinsen *et al*., 2018).

### Inclusion criteria

The full search yield was reviewed for inclusion by two independent reviewers (AdG/JU) on the basis of title and abstract. Both reviewers assessed full-texts of the remaining articles. Discrepancies were resolved by discussion, and remaining queries were discussed with a third reviewer (MS). The following inclusion criteria had to be met: *Population –* Arabic-speaking adults with no restrictions on setting. *Index test –* brief self-report questionnaires in Arabic on psychological distress, with no restrictions in terms of administration mode or administrator. We defined ‘brevity’ as 25 items or less, based on commonly used screening instruments (e.g. HSCL-25). We did not base our definition on, e.g. ‘time of administration’, given that time to complete a measure might vary among groups and literacy levels. *Reference standard* – a diagnosis made through a structured clinical interview or by a clinician based on the criteria of the Diagnostic and Statistical Manual of Mental Disorders (DSM) (American Psychiatric Association, 2013) or International Statistical Classification of Diseases and Related Health Problems (ICD) (WHO, 2019). *Outcome* – any CMD. CMDs refer to DSM/ICD diagnoses of anxiety, depressive (excluding bipolar), and stress-related disorders. Anxiety disorders include generalized anxiety disorder (GAD), panic disorder, phobia, agoraphobia, or social anxiety disorder. PTSD and acute stress disorder are included (as anxiety disorders in DSM-IV or as trauma- and stress-related disorders in DSM-5). We excluded papers in which the diagnosis was based on a questionnaire, observation checklist, chart review, or self-reported diagnosis. We also excluded studies that did not provide data to calculate sensitivity/specificity.

### Data extraction

Data were extracted independently from each study by two reviewers (AdG/IS) using a coding scheme (The Cochrane Collaboration, 2020). Extracted data included study design (design and study dates), participant characteristics (eligibility criteria, setting, sample size, age, gender, nationality, and comorbidities), index test characteristics (description, time points, mode of administration, setting, translation, scale properties, and psychometric properties), reference test characteristics (description, time points, mode of administration, blinding, setting, translation, prevalence, and psychometric properties), and relevant outcomes measured (target condition, thresholds with corresponding diagnostic accuracy properties, i.e. sensitivity, specificity, area under the receiver-operating characteristic (ROC) curve (AUC), PPV and NPV, and data to generate 2 × 2 tables). Discrepancies were resolved by discussion.

### Quality assessment

Risk of bias was independently assessed by two reviewers (AdG/IS) using the quality assessment tool of diagnostic accuracy studies (QUADAS-2) (Whiting *et al*., 2011). QUADAS-2 is a generic set of criteria consisting of four key domains: patient selection, index test, reference standard, and flow of patients through the study and timing of the index test and reference standard. Signaling questions are included to judge risk of bias across all domains (Whiting *et al*., 2011). We added three items to account for biases specific to the use of (semi-)structured clinical interviews. These extra items concerned (1) whether studies used a semi-structured interview *v.* clinician diagnosis (domain 3), (2) whether data on interviewer variation (e.g. inter-rater reliability) for the (semi)structured interview fell within an acceptable range (domain 3), and (3) whether all participants received a reference standard (domain 4). See online Supplementary Appendix 3 for item specifications.

### Data synthesis and statistical analysis

We provided a narrative synthesis structured around the type of index test (i.e. questionnaire) and type of outcome. For every study, we tabulated the questionnaire, reported cut-off scores and outcome measures. In this review, we present cut-off scores as rounded numbers (e.g. ‘5’), whereby individuals are considered positive cases if they have that score at minimum (e.g. 5 or above). Meta-analysis was performed when at least three studies with a comparable outcome for a specific questionnaire were included. Multiple thresholds were modelled for studies reporting a range of cut-off scores (Steinhauser *et al*., 2016) using the diagmeta package (Rucker *et al*., 2020) in R v3.6.1 (R Core Team, 2019). This approach incorporates the following issues relevant for diagnostic reviews: (1) imprecision by which the sensitivity or specificity has been measured within each study, (2) variation beyond chance in the sensitivity and specificity between studies, and (3) correlation that might exist between sensitivity and specificity. It also estimates the sensitivity and specificity for a range of cut-off scores and determines the optimal threshold, based on the cut-off with the highest combination of sensitivity and specificity using the Youden index. We plotted the estimates of sensitivity and specificity for each reported cut-off and the optimal threshold of all studies in the meta-analysis in ROC space.

## Results

### Study inclusion and characteristics of included studies

**Fig. 1.**
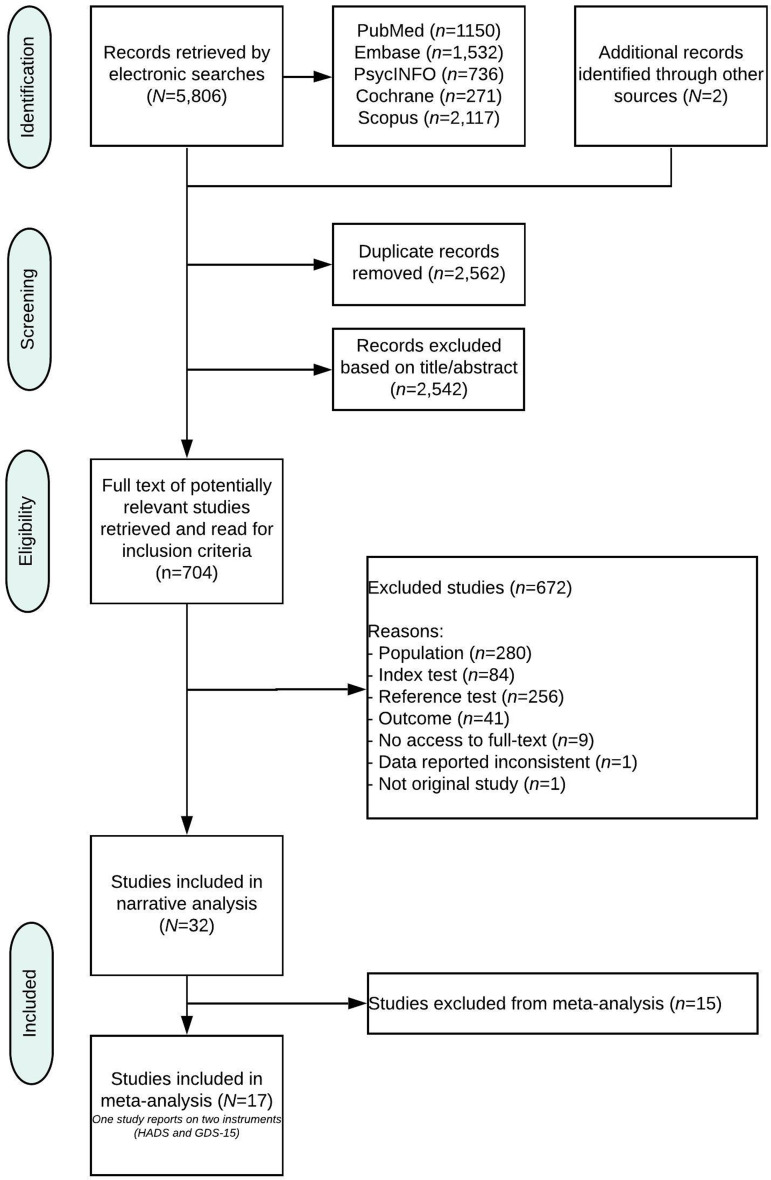
PRISMA-DTA Flow-chart.

The search yielded 3246 unique references (Fig. 1). Of these, 704 were identified as potentially relevant based on title/abstract screening. The full-text articles were obtained and assessed for inclusion. Thirty-two studies reporting on 30 unique datasets met the inclusion criteria. Of those, 17 studies were eligible for meta-analysis.

Seventeen different questionnaires on depression, anxiety, PTSD, and general distress were identified (Table 1). The number of items ranged from 5 to 25. Online Supplementary Appendix 4 provides a brief description of each questionnaire.

**Table 1.** Study Characteristics

Index test	Study	Study setting	Population	Sample size, *N*	Gender, % male	Age, *M*	Target condition	Reference test	Optimal/ †pre-defined cut-off [range reported]	Sensitivity	Specificity	PPV	NPV	AUC [95% CI]	Administration reference test; index test	Translation index test	Other psychometric properties index test
*Depression*																	
EPDS	Ghubash et al., 1997	UAE, clinic	Post-partum women	95	0	28.6	MDD	PSE	10† 12† [10, 12]	91% 73%	84% 90%	44% 50%	99% 96%	N/R	N/R; N/R	Own translation using back-translation by bilingual psychiatrists	Cronbach's alpha = 0.84; Split-half reliability = 0.82
EPDS	Barnett et al., 1999	Australia, clinic	Pregnant women (Arabic speaking migrants)	98	0	N/R	MDD	DIS	10† [8–13]	77.8%	80.2%	29.2%	N/R	N/R	Lay-interviewer; Interviewer (part) (partly in English)	Own translation using back-translation followed by pilot-testing, involvement of focus groups at each stage consisting of bilingual ethnic health workers.	N/R
EPDS	Agoub et al., 2005	Morocco, clinic	Post-partum women	144	0	30.3	MDD	MINI	12 [10–13]	92%	96%	86%	N/R	N/R	N/R; Interviewer (part)	Ghubash et al (1997)	N/R
EPDS	El-Hachem et al., 2014	Lebanon, clinic	Post-partum women	149	0	31.7	MDD	Clinician diagnosis (DSM-IV-TR) + EPDS >8	7 [5–12]	89.5%	47.7%	N/R	N/R	.82 [.72–.92]	Clinician; Self	N/R	N/R
EPDS	Khalifa et al., 2015	Sudan, clinic	Pregnant women	40	0	N/R	MDD	MINI	12† [1–15]	88.9%	81.8%	33.3%	98.8%	.89 [.78–.99]	Clinician; Interviewer (all)	N/R	N/R
EPDS	Naja et al., 2019	Qatar, clinic	Pregnant women	128	0	28.8	MDD	MINI	13 [8–14]	87%	90%	75%	N/R	.95 [.91–.99]	Clinician; Self	Own translation using back-translation by bilingual clinicians, discussion in a panel, and a pilot test of the questionnaire on a sample of *n* = 20 pregnant women.	Cronbach's alpha = 0.87; EPDS with BDI-II *r* = .6
EPDS	Shaheen et al., 2019	Saudi Arabia, clinic	Fathers	57 (sub-sample of 290)	100	35.0 (based on full *N*)	MDD	Clinician diagnosis (DSM 5)	9† [5–13/14]	77.8%	81.3%	N/R	N/R	.81	Clinician; Self	Ghubash et al (1997)	N/R
PHQ-9	Becker et al., 2002	Saudi Arabia, clinic	Primary care patients	173	44.5	N/R	Depression	SCID-R	3† [3 using diagnostic scoring system]	62%	95%	N/R	N/R	N/R	Clinician; Interviewer (part)	Own translation using back-translation by clinicians	N/R
PHQ-9	Hobfoll et al., 2011	Israel, community	Palestinian adults	75 (sub-sample of *N* = 150)	N/R	N/R	MDD	CIDI	5 (incl. depressed mood or lack of interest)† [5]	76%	96%	90%	89%	N/R	Interviewer (all)	N/R	N/R
PHQ-9	Sawaya et al., 2016	Lebanon, clinic	Psychiatric outpatients	176	46.4	35.6	Any mood disorder	Clinician diagnosis (DSM-IV)	10† [10]	77%	46%	N/R	N/R	.70	Clinician; Self	Own translation using back-translation by psychologists + discussion	Cronbach's alpha = 0.88
PHQ-9	Alzahrani et al., 2020	Saudi Arabia, clinic	Cancer patients	407	42.8	49.1	MDD	MINI	9 [5–10]	88.3% [76.8–94.8]	80.1% [75.4–84.1]	43.4%	97.5%	.91 [.88–.95]	Clinician; Self	Use of AlHadi et al. (2017)	Cronbach's alpha = .80
GDS-15	Chaaya et al., 2008	Lebanon, community & clinic	Community-dwelling elderly and primary care outpatients	105	25.0	69.8	MDD and Dysthymia	Clinician diagnosis (DSM-IV)	8 [5–9]	83%	91%	89%	87%	.89 [.82–.96]	Clinician; Interviewer (part)	Own translation using back-translation by a translator and psychiatrists + discussion + piloted among *n* = 10 older adults	Cronbach's alpha = 0.83, Spearman's correlation (test-retest, *n* = 38, 7 days interval) = 0.79
GDS-15	Hashim, 2018	Iraq, community & clinic	Elderly	279	49.5	71.8	MDD	Clinician diagnosis (ICD-10)	6 [6]	83.8%	90.6%	93.5%	77.4%	N/R	Clinician; Interviewer (part)	Own translation using back-translation by psychologists/psychiatrists	N/R
GDS-15	Karam et al., 2018	Lebanon, clinic	Psychiatric patients	57 (sub-sample of *N* = 132)	34.0	81.9	MDD	SCID-I	7 [6–7]	80%	87%	N/R	N/R	.90	Clinician; N/R	Own translation using back-translation by psychologists/psychiatrists + panel discussions	Cronbach's alpha = 0.84; Correlation GDS with other scales for sub-sample n = 57 *r* = 0.74 (Cornell Scale for Depression in Dementia) & 0.87 (HADS)
BDI-II	Naja et al., 2019	Qatar, clinic	Pregnant woman	128	0	28.8	Antenatal depression	MINI	19 [18–24]	96%	73%	54%	N/R	.91 [.86–.96]	Clinician; Self	Own translation using back-translation by bilingual clinicians, discussion in a panel, and a pilot test of the questionnaire in a sample of *n* = 20 pregnant women.	Cronbach's alpha = 0.90; EPDS with BDI-II *r* = .6; ICC *r* (based on pilot sample *n* = 20) = 0.59
CES-D	Ghubash et al., 2000	UAE, community	Medical students	30	0	N/R	Depression	SCID + self-evaluation of depression	21 [0–46]	82%	83%	N/R	N/R	.84	N/R; Self	Own translation using back-translation by bilingual psychiatrists and senior medical students, and a pilot test of the questionnaire in a medical student sample (pilot *n* = N/R).	Cronbach's alpha = 0.88; split-half *r* = 0.83; ICC *r* (2 weeks interval) = 0.69
MDI	Fawzi et al., 2012	Egypt, clinic	Depressed outpatients and healthy controls	100	39.0	N/R	Any mood disorder	SCID-I	5, incl. depressed mood or decreased interest† [5]	88.4%	78.9%	76%	90%	N/R	Clinician; Self	Own translation using back-translation by psychiatrists and non-clinical translators + discussion + pilot test of the questionnaire among *n* = 5	Cronbach's alpha = 0.91; ICC *r* (2 weeks interval) = 0.98
AES	Al-Adawi et al., 2004	Oman, clinic	TBI patients	80	66.3	31.0	Depression	CIDI	23 [18–72]	64.5%	62.8%	N/R	N/R	N/R	N/R; Interviewer (all)	Own translation using back-translation by experienced staff members	Inter-rater agreement *r* = 0.86
WHO-5	Sibai et al., 2009	Lebanon, community & clinic	Community-dwelling elderly and primary care outpatients	105	24.8	69.8	Depression	Clinician diagnosis (DSM-IV)	12 [12–16]^a^	78.3%	82.8%	78.7%	82.5%	.84 [.754–.920]	Clinician; Interviewer (part)	Own translation using back-translation by professional translators + discussed with psychiatrists + piloted among *n* = 10 older adults	Cronbach's alpha = 0.88, Spearman's correlation (test-retest, *n* = 38, 7 day-interval) = 0.73
PSST	Mahfoud et al., 2019	Qatar	Primary care patients	179	0	32.1	Premenstrual dysphoric disorder	MINI	≥1 of #1–4 is severe & ≥4 of #1–14 are moderate to severe & ≥1 of A-E is severe†	26.7%	95.6%	85.2%	58.3%	N/R	Clinician; Interviewer (all)	Own translation using back-translation by bilingual psychiatrist + piloted among *n* = 20 women	Cronbach's alpha = 0.92, Kappa (test-retest, *n* = 21, 7 day-interval) = 0.25
*Anxiety*																	
GAD-7	Sawaya et al., 2016	Lebanon, clinic	Psychiatric outpatients	176	46.4	35.6	Anxiety (GAD, panic disorder, phobia, and anxiety NOS)	Clinician diagnosis (DSM-IV)	10† [10]	57%	53%	N/R	N/R	.57	Clinician; Self	Own translation using back-translation by psychologists + discussion	Cronbach's alpha = 0.95
Anxiety modules PHQ (Panic/GAD)	Becker et al., 2002	Saudi Arabia, clinic	Primary care patients	173	44.5	N/R	Panic disorder	SCID-R	7† [7]	47%	96%	N/R	N/R	N/R	Clinician; Interviewer (part)	Own translation using back-translation by clinicians	Other psychometric properties: N/R
							GAD		4† [4]	37%	96%	N/R	N/R	N/R			
*Combined depression/anxiety*																	
HADS	El-Rufaie & Absood, 1995	UAE, clinic	Primary care patients	217	36.4	33 (median)	Anxiety	CIS (+OSR)	HADS-A: 7 (for economic reasons) [5–12]	78.0%	80.7%	N/R	N/R	N/R	Clinician; N/R	Adaptation of El-Rufaie et al. (1988) using back-translation by a bilingual psychiatrist + discussion	Cronbach's alpha = 0.78 (anxiety) & 0.88 (depression); Inter-rater reliability? Kappa range = 0.27–0.59 (anxiety) & 0.36–0.69 (depression)
							Depression		HADS-D: 4 [2–10]	81.1%	85.5%	N/R	N/R	N/R			
HADS	Al-Adawi et al., 2007	Oman, clinic	TBI patients	68	69.1	N/R	Anxiety	CIDI (ICD-10)	HADS-A: 5 [1–11]	61.8%	61.8%	N/R	N/R	.53	N/R; Interviewer (all)	Own translation using back-translation by ‘experienced staff members’	Cronbach's alpha = 0.95
							Depression		HADS-D: 4 [1–11]	53.8%	75.9%	N/R	N/R	N/R			
HADS	Al-Asmi et al., 2012	Oman, clinic	Patients with epilepsy	150	55.3	28.4	Any anxiety disorder	CIDI	HADS-A: 8 [1–18]	84.85%	91.25%	N/R	N/R	.95	N/R; Interviewer (all)	Own translation using back-translation by ‘experienced staff members’	N/R
							MDD		HADS-D: 8 [0–13]	87.50%	99.09%	N/R	N/R	.99			
HADS	Karam et al., 2018	Lebanon, clinic	Psychiatric patients	57	34	81.9	Anxiety	SCID-IV	HADS-A: 6 [6]	85%	83%	N/R	N/R	.92	Clinician; N/R	Own translation using back-translation by psychologists/psychiatrists + panel discussions	N/R
							MDD		HADS-D: 6 [6]	90%	70%	N/R	N/R	.84			
HSCL-25	Mahfoud et al., 2013	Lebanon, community	Community sample	153	0	36.2	Anxiety	MINI	HADS-A: 2.00 [1.75–2.30]	84%	59%	39%	92%	.75 [.67–.84]	Clinician; Interviewer (all)	Own translation using back-translation by psychiatrist/psychologist + discussion + pilot tested among *n* = 14 students	Cronbach's alpha = 0.91 (whole scale) & 0.85 (anxiety) & 0.88 (depression)
							Depression		HADS-D: 2.10 [1.75–2.30]	82%	70%	60%	87%	.85 [.78–.91)			
PCAD	El-Rufaie et al., 1997	UAE, clinic	Primary care patients	123	65	34 (median)	Depression and/or anxiety	Standardized clinical interview with 4-point scale rating for anxiety and depressive states (0 = non-case - 3 = severe)	8 [3–11]	81.8%	77.2%	N/R	N/R	N/R	Clinician; Interviewer (part)	Own translation using back-translation by psychiatrists + discussion	Cronbach's alpha = 0.91
*PTSD*																	
SPTSS	Caspi et al., 2007	Israel, community	Bedouin veterans	317	100	30.4	PTSD	SCID (DSM-IV) (Hebrew version)	6 [3–6]	89%	89%	58%	98%	.95 [.92–.97]	Lay-interviewer; Interviewer (all)	Own translation from English into Hebrew, and then from Hebrew into Arabic.	N/R
*Psychological distress*																	
SRQ-20	Climent et al., 1989	Sudan, clinic	Primary care patients and healthy controls	63	N/R	N/R	Mental disorders	PSE (short version)	4† [4]	N/R (we calculated: 92.9%)	N/R (we calculated: 95.2%)	N/R	N/R	N/R	Clinician; Interviewer (all)	Own translation from English to Arabic.	N/R
SRQ-20	El-Rufaie & Absood, 1994	UAE, clinic	Primary care patients	217	36.4	33 (median)	Any psychiatric diagnosis according to the mental disorders section of ICD-9	CIS (+OSR) + clinical judgement (ICD-9)	Whole scale: 6 [6]	78.3%	75.2%	54.7%	90.1%	N/R	Clinician; N/R	Adaptation of existing translation (reference N/R)	N/R
									Somatic questions: 3 [3]	75%	68.8%	N/R	N/R	N/R			
									Psychological questions: 3 [3]	85%	68.2%	N/R	N/R	N/R			
SRQ-20	Al-Subaie et al., 1998	Saudi Arabia, clinic	Patients referred for endoscopy	292	56.8	35.0	All affective, anxiety and somatoform disorders	Clinician diagnosis (DSM III-R)	7 [5–14]	93%	70%	75%	91%	N/R	Clinician; Interviewer (part)	N/R	Cronbach's alpha = 0.81
SRQ-20	Al-Arabi et al., 1999	Saudi-Arabia, clinic	Primary care patients with diabetes	49 (sub-sample of *N* = 226)	46.0	51.3	Mental disorders	Clinician diagnosis (DSM-IV)	10 [10]	70.6%	71.9%	57.1%	82.1%	N/R	Clinician; Interviewer (all)	N/R	Correlation between SRQ and Somatization Subscale of the RADS *r* = 0.74; Inter-rater reliability: mean Kappa (*n* = 68, 50% diabetic patients) = 0.84
SRQ-20	Llosa et al., 2017	Lebanon, community	Refugees/ migrants	55 (sub-sample of *N* = 748)	49.1	39.0	CMD; excl. substance use/eating/antisocial personality disorders	MINI	6† [1, 4, 6–7]	100%	82.5%	14.3%	100%	.91	Clinician; Interviewer (all)	N/R	Cronbach's alpha = 0.87
SRQ-20 + psychosis item	Alsuwaida & Alwahhabi, 2006	Saudi Arabia, clinic	Patients with end-stage renal disease on hemodialysis	26	58.0	48.1	MDD	Clinician diagnosis (DSM-IV)	13 [6–18	100%	83%	50%	N/R	.96	Clinician; N/R	N/R	N/R
GHQ-12	El-Rufaie & Daradkeh, 1996	UAE, clinic	Primary care patients	157	55.4	28.7	Any psychiatric diagnosis	CIS (+OSR)	13 [13]	83%	80%	87%	N/R	N/R	Clinician; Interviewer (all)	Own translation using back-translation by psychiatrists + discussion	N/R

a A lower score indicates less wellbeing, and participants with a cut-off score of 12 *or below* are considered case positives; AUC = area under the Receiver Operating Curve; CI = Confidence Interval; PPV = positive predictive value; NPV = negative predictive value; EPDS = Edinburgh Postnatal Depression Scale; PHQ-9 = Patient Health Questionnaire; GDS-15 = Geriatric Depression Scale; BDI-II = Beck Depression Inventory; CES-D = Center for Epidemiological Studies Depression Scale; MDI = Major Depression Inventory; AES = Apathy Evaluation Scale; WHO-5 = WHO Well-being Index; PSST = Premenstrual Symptoms Screening Tool; GAD-7 = Generalized Anxiety Disorder-7; HADS = Hamilton Anxiety and Depression Scale; HSCL-25 = Hopkins Symptoms Checklist; PCAD = Primary Care Anxiety and Depression; SPTSS = Screen for Posttraumatic Stress Symptoms; SRQ-20 = Self-Reporting Questionnaire; GHQ-12 = General Health Questionnaire

One study was conducted among a sub-sample of Arabic-speaking migrants in Australia (Barnett *et al*., 1999), while all other studies (*n* = 31) were conducted in Arab countries. Participants (*N* = 4042; range 26–407), with mean age range 28–82 years, were selected from clinical settings (*n* = 21, 65.6%), community settings (*n* = 5, 15.6%), or both (*n* = 4, 12.5%). Nine (28.1%) studies included only women, two (6.3%) only men, 17 (53.1%) mixed samples, and two (6.9%) did not report gender (6.9%). None of the questionnaires were locally developed, but all were translations of English-language instruments: in the majority of studies, questionnaires (*n* = 20, 62.5%) were locally translated, five (15.6%) used/adapted already existing translations, and seven (21.9%) did not report on translation. In 20 studies (62.5%), questionnaires were administered by interviewers.

Twenty-two studies (68.7%) used a (semi-)structured clinical interview as reference standard. Seven studies used the Mini International Neuropsychiatry Inventory (MINI), five the Structured Clinical Interview for DSM (SCID), four the Composite International Diagnostic Interview (CIDI), three the Clinical Interview Schedule (CIS), two the Present State Examination (PSE), and one the Diagnostic Interview Schedule (DIS). These (semi-)structured interviews were conducted by a clinician (*n* = 13) or lay-interviewer (*n* = 3); six studies did not report on the type of interviewer. In the other 10 studies (31.3%), a clinician diagnosis according to the DSM/ICD was made. El-Hachem *et al*. (2014) combined the clinical interview with (readministration of) the index test.

### Results of the systematic review

Nine depression-specific questionnaires were compared to a depression diagnosis (Table 1). The sensitivity in seven studies evaluating the EPDS ranged from 73% to 92%; its specificity ranged from 48% to 96%. The nine-item Patient Health Questionnaire (PHQ-9) was evaluated in four studies, with sensitivity ranging from 62% to 88%, and specificity from 46% to 96%. Three studies evaluated the Geriatric Depression Scale (GDS-15), with sensitivity ranging from 80% to 84%, and specificity from 87% to 91%. The other depression-specific instruments were evaluated by single studies. The Beck Depression Inventory-II (BDI-II) had a sensitivity of 96% and a specificity of 73%, the Center for Epidemiologic Studies Depression Scale (CES-D) had a sensitivity of 82% and a specificity of 83%, the Major Depression Inventory (MDI) had a sensitivity of 88% and a specificity of 79%, the Apathy Evaluation Scale (AES) had a sensitivity of 65% and a specificity of 63%, and the five-item WHO Well-being Index (WHO-5) had a sensitivity of 78% and a specificity of 83%. The Premenstrual Symptoms Screening Tool (PSST) was compared to a diagnosis of premenstrual dysphoric disorder and had a sensitivity of 27% and a specificity of 96%.

We found two anxiety-specific questionnaires. One study compared the seven-item Generalized Anxiety Disorder (GAD-7) to any anxiety disorder, with a sensitivity of 57% and a specificity of 53%, and one study compared the PHQ modules panic, with a sensitivity of 47% and a specificity of 96%, and GAD, with a sensitivity of 37% and a specificity of 96%, to corresponding DSM-IV criteria.

We found three instruments targeting combined anxiety/depression that were compared to a diagnosis of anxiety and/or depression. The HADS was evaluated in four studies. The sensitivity of the anxiety subscale ranged from 62% to 85%, and its specificity from 62% to 91%. The sensitivity range of the depression subscale was 54–90%, and specificity range 70–99%. One study evaluated the HSCL-25. The sensitivity of the anxiety subscale was 84%, and its specificity 59%. The sensitivity of the depression subscale was 82%, and its specificity 70%. The Primary Care Anxiety and Depression scale was evaluated in one study, which found a sensitivity of 82% and a specificity of 77%.

We found one instrument targeting PTSD symptoms. The Screen for Posttraumatic Stress Symptoms (SPTSS) had a sensitivity of 89% and a specificity of 89% compared to a PTSD diagnosis.

Lastly, we identified two general distress instruments that were compared to a diagnosis of any CMD. The 20-item Self-Reporting Questionnaire (SRQ-20) was investigated in six studies, of which one study also included a psychosis item. The sensitivity range was 71–100%; the specificity range 70–95%. The 12-item General Health Questionnaire (GHQ-12) was evaluated in one study and had a sensitivity of 83% and a specificity of 80%.

Online Supplementary Appendix 5 presents a visual representation for all instruments for which we included at least three studies.

### Quality of studies

The QUADAS-2 results are evaluated at item-level and do not incorporate an overall quality score (Table 2). Eleven studies scored high risk of bias on one domain, 14 on two domains, three on three domains, and none on all four domains. Four studies did not score high risk on any of the domains.

**Table 2:** Risk of bias (QUADAS-2)

		Risk of bias				Applicability concerns		
Index test	Study	Patient selection	Index test	Reference standard	Flow and timing	Patient selection	Index test	Reference standard
*Depression*								
EPDS	Agoub *et al*. (2005)	☺	☹	☹	☺	☺	☺	☺
EPDS	Barnett *et al*. (1999)	☺	☹	☹	☺	☺	☹^a^	☺
EPDS	El-Hachem *et al*. (2014)	☺	☹	☹	☹	☺	☺	☺
EPDS	Ghubash *et al*. (1997)	☺	☺	☹	☹	☺	☺	☺
EPDS	Khalifa *et al*. (2015)	☺	☺	☺	☹	☺	☺	☺
EPDS	Shaheen *et al*. (2019)	☹	☺	☹	☹	☺	☺	☺
EPDS	Naja *et al*. (2019)	☺	☹	☺	☺	☺	☺	☺
PHQ-9	Becker *et al*. (2002)	☺	☺	?	☺	☺	☺	☺
PHQ-9	Hobfoll *et al*. (2011)	☺	☺	?	☺	☺	☺	☺
PHQ-9	Sawaya *et al*. (2016)	☺	☺	☹	☺	☺	☺	☺
PHQ-9	Alzahrani *et al*. (2020)	☺	☹	?	☺	☺	☺	☺
GDS-15	Chaaya *et al*. (2008)	?	☹	☹	☺	☺	☺	☺
GDS-15	Hashim (2018)	?	?	☹	☺	?	☺	☺
GDS-15	Karam *et al*. (2018)	☺	☹	☺	☹	☺	☺	☺
BDI-II	Naja *et al*. (2019)	☺	☹	☺	☺	☺	☺	☺
CES-D	Ghubash *et al*. (2000)	☹	☹	?	☺	☺	☺	☺
MDI	Fawzi *et al*. (2012)	☹	☹	?	☺	☺	☺	☺
AES	Al-Adawi *et al*. (2004)	☹	☺	☺	☺	☺	☺	☺
WHO-5	Sibai *et al*. (2009)	?	☹	☹	☺	☺	☺	☺
PSST	Mahfoud *et al*. (2019)	☹	☺	☺	☺	☺	☹	☺
*Anxiety*								
GAD-7	Sawaya *et al*. (2016)	☺	☺	☹	☺	☺	☺	☺
Anxiety modules PHQ (Panic and GAD)	Becker *et al*. (2002)	☺	☺	?	☺	☺	☺	☺
*Combined depression/anxiety*								
HADS	El-Rufaie and Absood (1995)	☺	☹	☺	☺	☺	☺	☺
HADS	Al-Adawi *et al*. (2007)	☹	☺	☺	☺	☺	☺	☺
HADS	Al-Asmi *et al*. (2012)	☹	☹	☺	☺	☺	☺	☺
HADS	Karam *et al*. (2018)	☺	☹	☺	☹	☺	☺	☺
HSCL-25	Mahfoud *et al*. (2013)	☹	☹	☺	☺	☺	☺	☺
PCAD	El-Rufaie *et al*. (1997)	☺	☹	☹	☺	☺	☺	☺
*PSTD*								
SPTSS	Caspi *et al*. (2007)	☹	☹	?	☺	☺	☺	☺
*Psychological distress*								
SRQ-20	Climent *et al*. (1989)	☺	?	☺	?	☺	☺	☺
SRQ-20	El-Rufaie and Absood (1994)	☺	☹	☺	☺	☺	☺	☺
SRQ-20	Al-Subaie *et al*. (1998)	☺	☹	☹	☺	☺	☺	☺
SRQ-20	Al-Arabi *et al*. (1999)	☺	☹	☹	?	☺	☺	☺
SRQ-20	Llosa *et al*. (2017)	☺	☺	☺	?	?	☺	☺
SRQ-20 + psychosis item	Alsuwaida and Alwahhabi (2006)	☹	☹	☹	☺	☺	☺	☺
GHQ-12	El-Rufaie and Daradkeh (1996)	☺	☹	☺	☺	☺	☺	☺

Quality of studies rated as ☺ = low risk, ☹ = high risk, ? = unclear. EPDS, Edinburgh Postnatal Depression Scale; PHQ-9, Patient Health Questionnaire; GDS-15, Geriatric Depression Scale; BDI-II, Beck Depression Inventory; CES-D, Center for Epidemiological Studies Depression Scale; MDI, Major Depression Inventory; AES, Apathy Evaluation Scale; WHO-5, WHO Well-being Index; PSST, Premenstrual Symptoms Screening Tool; GAD-7, Generalized Anxiety Disorder-7; HADS, Hamilton Anxiety and Depression Scale; HSCL-25, Hopkins Symptoms Checklist; PCAD, Primary Care Anxiety and Depression; SPTSS, Screen for Posttraumatic Stress Symptoms; SRQ-20, Self-Reporting Questionnaire; GHQ-12, General Health Questionnaire.

a Part of Arabic-speaking sub-sample completed test in English.

Risk of bias for *Patient Selection* was low in the majority of studies. Studies scored high risk if a case-control design was used (Fawzi *et al*., 2012) if participants were not recruited at random (Ghubash *et al*., 2000; Caspi *et al*., 2007; Mahfoud *et al*., 2013), or in case of inappropriate exclusions (Al-Adawi *et al*., 2004, 2007; Alsuwaida and Alwahhabi, 2006; Al-Asmi *et al*., 2012; Mahfoud *et al*., 2013, 2019; Shaheen *et al*., 2019). Risk was unclear in three studies, because the method of recruitment was unclear (Chaaya *et al*., 2008; Sibai *et al*., 2009; Hashim, 2018).

Studies were rated high risk for *Index Test*, because the questionnaire was completed after the reference standard and/or because the threshold was not pre-defined (El-Rufaie and Absood, 1994, 1995; El-Rufaie and Daradkeh, 1996; El-Rufaie *et al*., 1997; Al-Subaie *et al*., 1998, 1999; Barnett *et al*., 1999; Ghubash *et al*., 2000; Agoub *et al*., 2005; Alsuwaida and Alwahhabi, 2006; Caspi *et al*., 2007; Chaaya *et al*., 2008; Sibai *et al*., 2009; Al-Asmi *et al*., 2012; Fawzi *et al*., 2012; Mahfoud *et al*., 2013; El-Hachem *et al*., 2014; Karam *et al*., 2018; Naja *et al*., 2019; Alzahrani *et al*., 2020).

Fourteen studies were rated high risk for *Reference Test*, because an unstructured clinician diagnosis rather than a semi-structured interview was used (El-Rufaie *et al*., 1997; Al-Subaie *et al*., 1998; Al-Arabi *et al*., 1999; Chaaya *et al*., 2008; Sibai *et al*., 2009; El-Hachem *et al*., 2014; Sawaya *et al*., 2016; Hashim, 2018; Shaheen *et al*., 2019), and/or because interviewers were not blinded (Agoub *et al*., 2005; Ghubash *et al*., 1997; Barnett *et al*., 1999). None of the studies reported interrater reliability.

In *Flow and Timing*, risk was high in eight studies, because of an inappropriate time interval between index and reference test (Ghubash *et al*., 1997), and/or because not all participants were included in the analysis (El-Hachem *et al*., 2014; Khalifa *et al*., 2015; Sawaya *et al*., 2016; Karam *et al*., 2018; Shaheen *et al*., 2019).

Supplementary data and clarification were provided for three studies (Becker *et al*., 2002; Alsuwaida and Alwahhabi, 2006; Al-Asmi *et al*., 2012) after correspondence with authors.

### Results of the meta-analysis

**Fig. 2.**
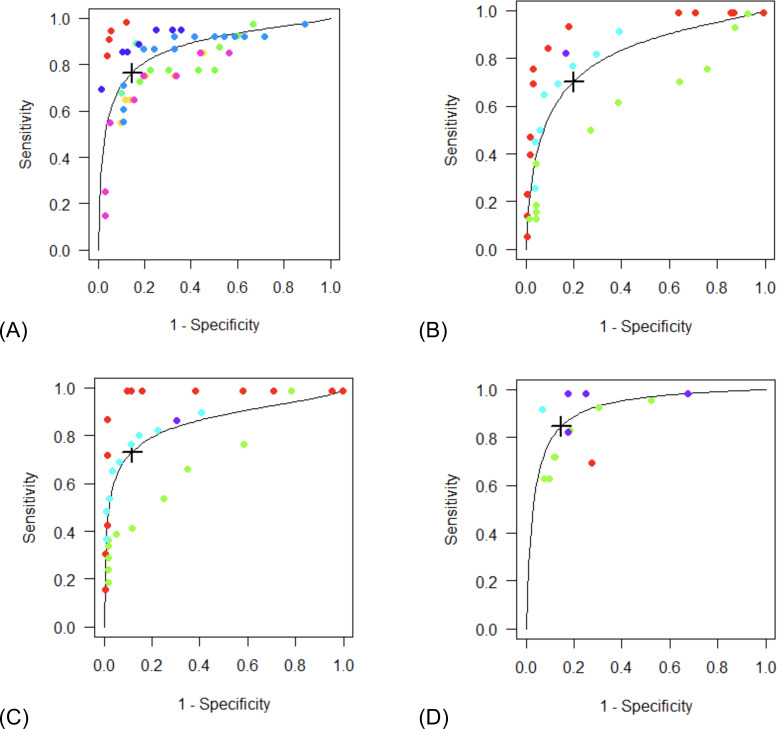
SROC plots for the EPDS (A), HADS-A (B), HADS-D (C) and SRQ-20 (D).

We meta-analyzed studies (if at least three per questionnaire) reporting on the same questionnaire and comparable target condition. Optimal thresholds for the EPDS, HADS anxiety and depression subscales (HADS-A and HADS-D), and SRQ-20 could be estimated. Two studies on the SRQ-20 were excluded from meta-analysis, because of missing data to calculate the 2 × 2 table (El-Rufaie and Absood, 1994), and because a 21-item version was used (Alsuwaida and Alwahhabi, 2006). We also performed meta-analysis on the GDS-15, but results were unreliable due to limited data and therefore only presented in online Supplementary Appendix 6. Pooled AUC statistics were >0.80 for all questionnaires. The summary operating points per questionnaire at different thresholds are provided in Table 3 and visually presented in summary ROC (SROC) plots in Fig. 2. We also included the Youden index and ROC/SROC curves, and 2 × 2 tables in online Supplementary Appendix 6.

**Table 3:** Summary operating points of sensitivity and specificity by questionnaire

Questionnaire	Cut-off	Studies *n*	Participants *n*	Sensitivity % (95% CI)	Specificity % (95% CI)	Pooled AUC (95% CI)^a^
EPDS	9	5	472	84.4 (72.9–91.5)	74.0 (65.7–80.8)	0.873 (0.791–0.930)
	10	7	711	80.9 (67.1–89.8)	80.1 (72.5–86.1)	
	**11^b^**	**6**	**616**	**76.9 (60.6–87.7)**	**85.2 (78.4–90.1)**	
	12	7	711	72.3 (53.6–85.4)	89.1 (83.3–93.0)	
	13	5	467	67.2 (46.4–82.9)	92.1 (87.2–95.2)	
HADS-A	5	3	431	86.4 (63.9–95.8)	52.5 (38.3–66.2)	0.813 (0.619–0.924)
	6	4	488	80.1 (53.1–93.5)	66.7 (53.1–78.0)	
	**7^c^**	**3**	**431**	**71.9 (41.9–90.1)**	**78.5 (67.3–86.6)**	
	8	3	431	61.9 (31.5–85.1)	86.9 (78.7–92.2)	
	9	3	431	50.7 (22.6–78.4)	92.3 (86.9–95.6)	
HADS-D	4	3	435	84.4 (65.4–940)	67.2 (45.4–83.5)	0.856 (0.701–0.940)
	5	3	435	79.3 (57.4–91.6)	80.0 (61.8–90.8)	
	**6^d^**	**4**	**492**	**73.0 (48.9–88.4)**	**88.6 (75.7–95.1)**	
	7	3	435	65.6 (40.3–84.3)	93.8 (85.7–97.4)	
	8	3	435	57.4 (32.3–79.2)	96.7 (92.0–98.7)	
SRQ-20	6	3	564	91.8 (86.3–95.3)	73.5 (42.5–91.3)	0.917 (0.876–0.945)
	7	2	347	89.3 (82.6–93.6)	79.2 (50.3–93.5)	
	**8^e^**	**1**	**292**	**86.0 (78.0–91.4)**	**83.9 (58.1–95.1)**	
	9	1	292	82.0 (72.6–88.6)	87.7 (65.4–96.4)	
	10	2	341	77.1 (66.2–85.2)	90.7 (72.0–97.4)	

EPDS, Edinburgh Postnatal Depression Scale; SRQ-20, Self-Reporting Questionnaire.

a We reported the 95% CI of the AUC for sensitivity given specificity.

b The model estimated an optimal threshold for the EPDS of 11.08 (sensitivity = 76.5% and specificity = 85.5%).

c The model estimated an optimal threshold for the HADS-A of 7.17 (sensitivity = 70.3% and specificity = 80.1%).

d The model estimated an optimal threshold for the HADS-D of 5.97 (sensitivity = 73.2% and specificity = 88.4%).

e The model estimated an optimal threshold for the SRQ-20 of 8.36 (sensitivity = 86.0% and specificity = 83.9%).

Bold values signifies the best cut-off.

Our model identified 11.08 as optimal threshold for the EPDS (*n* = 7); resulting in a practically relevant optimal cut-off score of 11, with a pooled sensitivity of 76.9% (95% confidence interval [CI] 60.6–87.7) and a specificity of 85.2% (95% CI 78.4–90.1).

The HADS-A model (*n* = 4) identified 7.17 as an optimal threshold, indicating a practically relevant cut-off score of 7 with a pooled sensitivity of 71.9% (95% CI 41.9–90.1) and a specificity of 78.5% (95% CI 67.3–86.6). The HADS-D model (*n* = 4) identified 5.97 as an optimal threshold, with 6 as the closest, practically relevant cut-off score, having a pooled sensitivity of 73.0% (95% CI 48.9–88.4) and a specificity of 88.6% (95% CI 75.7–95.1). CIs for the sensitivity/specificity estimates of the HADS subscales were wide, also illustrated by widely varying ROC curves in Fig. 2, indicating low discriminative ability.

Finally, the SRQ-20 model (*n* = 4) identified 8.36 as an optimal threshold, indicating a practically relevant cut-off score of 8 with a pooled sensitivity of 86.0% (95% CI 78.0–91.4) and a specificity of 83.9% (95% CI 58.1–95.1). The questionnaire's CIs associated with the pooled specificity were particularly wide.

## Discussion

Brief psychological screening instruments are commonly used in research and clinical practice for the measurement of symptom severity, but also as inexpensive, easy-to-administer tools for case-finding (Kagee *et al*., 2013; Olin *et al*., 2017). This systematic review and meta-analysis investigated the diagnostic performance of brief, Arabic-language screening instruments in detecting the symptoms of CMDs.

We synthesized the current evidence of 17 questionnaires, including instruments targeting depression, anxiety, general distress, and PTSD. A first finding is that, while the majority of studies reported on depression-specific questionnaires, the evidence for PTSD-specific instruments is limited. We must note, however, that we excluded several papers on the validity of PTSD screening tools in mixed-language populations (Söndergaard *et al*., 2003; Jakobsen *et al*., 2011; Ibrahim *et al*., 2018), since they did not separately report data on Arabic-speaking sub-samples. Another general finding is that we did not identify locally developed screening tools, and this review only synthesized evidence on Arabic translations of screeners originally developed in other settings.

The studies included in this review differed in many ways from each other. Studies varied with regard to target condition (e.g. major depressive disorder *v.* any mood disorder), population (e.g. pregnant women *v.* elderly), and setting (e.g. clinical sample in Sudan *v.* community sample in Lebanon). Although this review focused on Arabic-speaking populations, the global Arabic-speaking community cannot be considered as one monolithic cultural group with identical idioms of distress or manifestations of psychological distress (e.g. Hassan *et al*., 2016). Modern Standard Arabic (formal Arabic) is the only standardized form of written Arabic and is commonly understood among Arabic-speakers. Questionnaires in written form should thus be applicable across Arabic-speaking populations. However, in the majority of studies, questionnaires were administered by an interviewer, and thus read aloud. Even if questionnaires were written in formal Arabic, interviewers and participants may have communicated (or clarified) using their local dialects. Furthermore, most screening instruments were locally translated, and this might have introduced minor linguistic differences between translations. All but one study were conducted in Arabic countries, and covered Arabic-speaking populations in both high-income countries (e.g. Saudi Arabia) and LAMICs (e.g. Egypt).

Meta-analytic evidence was provided for the EPDS, HADS, and SRQ-20. Although AUCs were high, this statistic summarizes overall model performance over all possible thresholds. In practice, however, a specific threshold is used to discriminate between cases and non-cases, and determines the number of false-negative and false-positive cases. Thus, a single cut-off score may not perform as good as expected by overall test performance.

The present review found that a cut-off of 11 on the EPDS maximized combined sensitivity (76.9%)/specificity (85.2%). This threshold is lower compared to the original cut-off of 13 in English-speaking populations (Cox *et al*., 1987). A recent meta-analysis of individual participant data (IPDMA) on the EPDS also found that a threshold of 11 maximized combined sensitivity (81%)/specificity (88%) (Levis *et al*., 2020). Earlier reviews found the EPDS to be valid for non-English-speaking populations (Zubaran *et al*., 2010; Russell *et al*., 2020). The EPDS is one of the most frequently studied instruments in perinatal populations in LAMICs (Chorwe-Sungani and Chipps, 2017). Ali *et al*. (2016) conclude that the instrument generally performs well in LAMICs, while a systematic review in low- and lower-middle income countries, without Arabic-speaking samples, found that none of the studies had an accuracy of >80% on all three accuracy parameters (sensitivity/specificity/PPV) (Shrestha *et al*., 2016). The optimal cut-off score in our meta-analysis would miss almost a quarter of individuals with depression. Clinicians may therefore consider using a lower cut-off to identify potential cases for the purpose of triage (e.g. positive cases will be further assessed with a clinical interview). For example, a cut-off score of 9 would miss 15.6% of individuals with depression, but at the cost of screening 26.2% of non-cases as cases. However, in low-resourced settings where there is no capacity to assess all positive cases with a clinical interview, a high number of false positives (resulting from low specificity), is likely to overburden local health systems (Andersen *et al*., 2020). In these settings, a higher cut-off with improved specificity might be preferable.

We found substantial heterogeneity in the test performance of the HADS. A cut-off of 7 was optimal for the HADS-A based on maximized combined sensitivity (71.9%) and specificity (78.5%), and of 6 for the HADS-D (sensitivity: 73.0%/specificity: 88.6%). CIs for HADS were wide, indicating uncertainty about the estimated psychometric properties. In a recent IPDMA on the accuracy of the HADS-D to estimate depression prevalence, Brehaut *et al*. (2020) found the commonly used cut-off of 8 (‘doubtful cases’) significantly overestimated depression prevalence, while a cut-off of 11 (‘definite cases’) may either over- or underestimate depression prevalence. Ali *et al*. (2016) conclude that the HADS-A is an adequate screener in LAMICs, but reported strong to very strong validity for primary studies that used the English (with Yoruba) version of HADS-A, and weak to strong validity for other language versions (Portuguese and Chinese) (Ali *et al*., 2016). Based on our meta-analyses and in line with Brehaut *et al*. (2020), the evidence for the validity of the Arabic HADS is questionable.

The SRQ-20 as a screener for CMDs maximized combined sensitivity/specificity at a cut-off of 8 (86.0% and 83.9%, respectively). In other words, 14% of individuals with a disorder will remain undetected, while 16.1% of individuals without a disorder screen positive. The CIs for specificity were relatively wide. We therefore suggest that the SRQ-20 cut-off of 8 is useful for screening purposes to *rule out* the presence of any CMD, but that the questionnaire might be less reliable for *ruling in* because of uncertainty about the pooled specificity. A cut-off of 8 is commonly used (Harpham *et al*., 2003), although prior research has shown that optimal thresholds for the SRQ-20 differ considerably across settings, languages, cultures, and gender (e.g. Harding *et al*. 1980; Ventevogel *et al*. 2007). For example, a cut-off score of 6 gave the best sensitivity/specificity balance in two studies in low-resource primary care settings in Eritrea and South Africa. Both studies also found that performance improved among men by using an even lower cut-off (Van der Westhuizen *et al*., 2017; Netsereab *et al*., 2018).

This review has several strengths and limitations. A strength is that it provides researchers and clinicians working with Arabic-speaking populations with an overview of the validity of brief screening tools, and empirically grounded recommendations for thresholds. We provided the results of multi-threshold models, rather than bivariate models in which only one threshold per study can be pooled. In doing so, we were able to provide the pooled accuracy statistics at different cut-off scores, allowing researchers and clinicians to decide which threshold is most suitable (e.g. for epidemiological studies *v.* screening in stepped care).

A limitation of this paper concerns the wide range of reference standards used, including both (semi-)structured interviews and (unstructured) clinician diagnoses. Clinician diagnoses may be less reliable than (semi-)structured interviews (Segal and Williams, 2014). The literature, however, also highlights the limitations of structured interviews. For example, the MINI may overestimate the presence of mental disorders (Levis *et al*., 2018; Wu *et al*., 2020). Another limitation is related to the quality of studies, with 17 studies scoring high risk of bias on at least two QUADAS-2 domains. The majority of studies did not pre-specify a cut-off score, which may lead to overestimation of the accuracy estimates (Whiting *et al*., 2011). Furthermore, for some questionnaires, primary studies differed with respect to target condition and reported thresholds, due to which we could not meta-analyze those studies (e.g. PHQ-9). Due to low numbers of studies per questionnaire, we could not perform further subgroup analyses. Consequently, we included both antenatal and postnatal, as well as female-only and male-only samples in our meta-analysis on the EPDS, while these sub-samples may require different thresholds (Matthey *et al*., 2006; Gibson *et al*., 2009; Ali *et al*., 2016). We were also not able to investigate differences across Arabic-speaking populations (e.g. by country).

The clinical implications of this review are that a cut-off of 11 on the Arabic-language EPDS could be used as a screener for depression in perinatal populations to optimize a balance between sensitivity/specificity. For ruling out the presence of any CMD with the SRQ-20, we recommend using a cut-off score of 8. The evidence for the HADS to screen for depression and/or anxiety was not convincing as results were substantially heterogeneous.

This review also stresses the paucity of evidence on anxiety and PTSD screeners. Future studies are needed to investigate the diagnostic accuracy of questionnaires to detect anxiety and PTSD in Arabic-speaking populations given the amount of Arabic-speaking refugees at risk for developing stress-related disorders (Peconga and Høgh Thøgersen, 2020). According to our QUADAS-2 assessment, future studies can be improved by using semi-structured interviews as reference standard, such as the SCID, and report on the interrater reliability. We recommend pre-defining thresholds to prevent the overestimation of accuracy estimates.

## Conclusions

This review identified 17 brief questionnaires in the Arabic language that were investigated on diagnostic performance, with limited availability of evidence for PTSD instruments. The meta-analysis provided optimal cut-off scores for the EPDS, HADS, and SRQ-20.

## Data Availability

The data that support the findings of the meta-analysis are available in the online Supplementary material of this article.

## Supplementary material

For supplementary material accompanying this paper visit http://dx.doi.org/10.1017/gmh.2021.39. click here to view supplementary material
