# Supplementary material for: A systematic review and meta-analysis of diagnostic test accuracy studies of self-report screening instruments for common mental disorders in Arabic-speaking adults
Source: Glob Ment Health (Camb). 2021 Nov 23;8:e43. doi: 10.1017/gmh.2021.39 (PMC8679833; doi:10.1017/gmh.2021.39)
Supplement: Supplementary file 1 [file S205442512100039Xsup001.zip › Appendix 6. Graphs and 2x2 tables.docx]

**Supplementary material**

**Appendix 6**

| 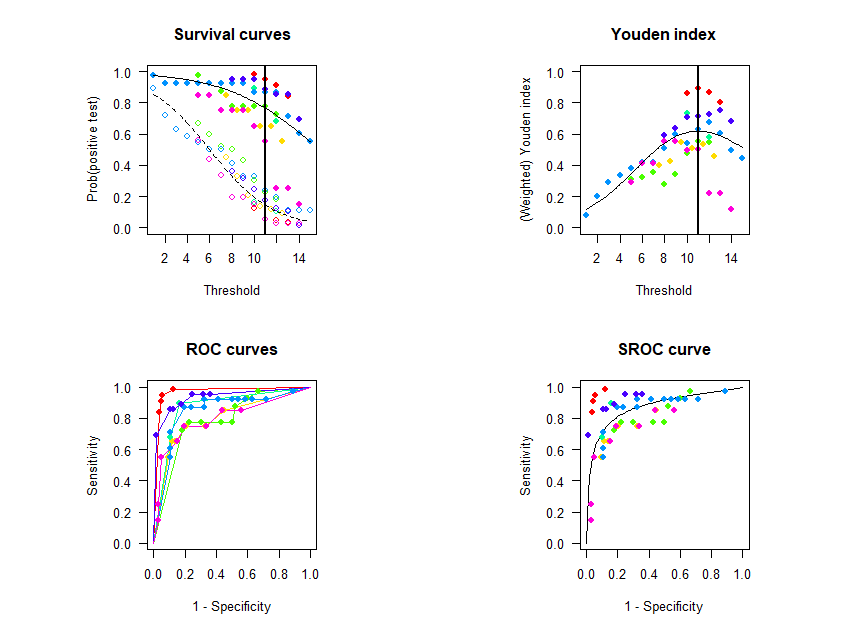 |
| --- |
| *Figure A6a.* Survival Curves, Youden Index, ROC and SROC Plots EPDS |

| Table A6a.  *2x2 Table for the EPDS* | | | | | | | | | | | | |
| --- | --- | --- | --- | --- | --- | --- | --- | --- | --- | --- | --- | --- |
|  |  | Reference standard | | | | Index test | | | 2x2 Table | | | |
| Study | Total, *N* | With diagnosis, *n* | Without diagnosis, *n* | Prevalence, % | Target Condition | Cut-off score | Sensitivity | Specificity | TP | FP | TN | FN |
| Ghubash et al. (1997) | 95 | 13 | 82 | 14.0 | MDD | 10 | 0.91 | 0.84 | 12 | 13 | 69 | 1 |
|  |  |  |  |  |  | 12 | 0.73 | 0.90 | 9 | 8 | 74 | 4 |
| Barnett et al. (1999) | 98 | 9 | 89 | 9.2 | MDD | 8 | 0.89 | 0.55 | 8 | 40 | 49 | 1 |
|  |  |  |  |  |  | 9 | 0.78 | 0.67 | 7 | 29 | 60 | 2 |
|  |  |  |  |  |  | 10 | 0.78 | 0.80 | 7 | 18 | 71 | 2 |
|  |  |  |  |  |  | 11 | 0.67 | 0.86 | 6 | 12 | 77 | 3 |
|  |  |  |  |  |  | 12 | 0.67 | 0.88 | 6 | 10 | 79 | 3 |
|  |  |  |  |  |  | 13 | 0.56 | 0.91 | 5 | 8 | 81 | 4 |
| Agoub et al. (2005) | 144 | 27 | 117 | 18.8 | MDD | 10 | 1 | 0.88 | 27 | 14 | 103 | 0 |
|  |  |  |  |  |  | 11 | 0.96 | 0.95 | 26 | 6 | 111 | 1 |
|  |  |  |  |  |  | 12 | 0.92 | 0.96 | 25 | 5 | 112 | 2 |
|  |  |  |  |  |  | 13 | 0.85 | 0.97 | 23 | 4 | 113 | 4 |
| El-Hachem et al. (2014) | 149 | 19 | 130 | 12.8 | MDD | 5 | 1 | 0.33 | 19 | 87 | 43 | 0 |
|  |  |  |  |  |  | 6 | 0.95 | 0.40 | 18 | 78 | 52 | 1 |
|  |  |  |  |  |  | 7 | 0.89 | 0.48 | 17 | 68 | 62 | 2 |
|  |  |  |  |  |  | 8 | 0.79 | 0.50 | 15 | 65 | 65 | 4 |
|  |  |  |  |  |  | 9 | 0.79 | 0.57 | 15 | 56 | 74 | 4 |
|  |  |  |  |  |  | 10 | 0.79 | 0.70 | 15 | 39 | 91 | 4 |
|  |  |  |  |  |  | 11 | 0.79 | 0.78 | 15 | 29 | 101 | 4 |
|  |  |  |  |  |  | 12 | 0.74 | 0.82 | 14 | 23 | 107 | 5 |
| Khalifa et al. (2015) | 40 | 18 | 22 | 45.0 | MDD | 1 | 1 | 0.09 | 18 | 20 | 2 | 0 |
|  |  |  |  |  |  | 2 | 0.94 | 0.27 | 17 | 16 | 6 | 1 |
|  |  |  |  |  |  | 3 | 0.94 | 0.36 | 17 | 14 | 8 | 1 |
|  |  |  |  |  |  | 4 | 0.94 | 0.41 | 17 | 13 | 9 | 1 |
|  |  |  |  |  |  | 5 | 0.94 | 0.46 | 17 | 12 | 10 | 1 |
|  |  |  |  |  |  | 6 | 0.94 | 0.50 | 17 | 11 | 11 | 1 |
|  |  |  |  |  |  | 7 | 0.94 | 0.50 | 17 | 11 | 11 | 1 |
|  |  |  |  |  |  | 8 | 0.94 | 0.59 | 17 | 9 | 13 | 1 |
|  |  |  |  |  |  | 9 | 0.94 | 0.68 | 17 | 7 | 15 | 1 |
|  |  |  |  |  |  | 10 | 0.89 | 0.68 | 16 | 7 | 15 | 2 |
|  |  |  |  |  |  | 11 | 0.89 | 0.77 | 16 | 5 | 17 | 2 |
|  |  |  |  |  |  | 12 | 0.89 | 0.82 | 16 | 4 | 18 | 2 |
|  |  |  |  |  |  | 13 | 0.72 | 0.91 | 13 | 2 | 20 | 5 |
|  |  |  |  |  |  | 14 | 0.61 | 0.91 | 11 | 2 | 20 | 7 |
|  |  |  |  |  |  | 15 | 0.56 | 0.91 | 10 | 2 | 20 | 8 |
| Naja et al. (2019) | 128 | 30 | 98 | 24.0 | MDD | 8 | 0.96 | 0.64 | 29 | 35 | 63 | 1 |
|  |  |  |  |  |  | 9 | 0.96 | 0.68 | 29 | 31 | 67 | 1 |
|  |  |  |  |  |  | 10 | 0.96 | 0.76 | 29 | 24 | 74 | 1 |
|  |  |  |  |  |  | 11 | 0.90 | 0.83 | 27 | 17 | 81 | 3 |
|  |  |  |  |  |  | 12 | 0.87 | 0.88 | 26 | 12 | 86 | 4 |
|  |  |  |  |  |  | 13 | 0.87 | 0.90 | 26 | 10 | 88 | 4 |
|  |  |  |  |  |  | 14 | 0.71 | 0.99 | 21 | 1 | 97 | 9 |
| Shaheen et al. (2019) | 57 | 9 | 48 | 16.0 | MDD | 5 | 0.89 | 0.44 | 8 | 27 | 21 | 1 |
|  |  |  |  |  |  | 6 | 0.89 | 0.56 | 8 | 21 | 27 | 1 |
|  |  |  |  |  |  | 7 | 0.78 | 0.67 | 7 | 16 | 32 | 2 |
|  |  |  |  |  |  | 8 | 0.78 | 0.81 | 7 | 9 | 39 | 2 |
|  |  |  |  |  |  | 9 | 0.78 | 0.81 | 7 | 9 | 39 | 2 |
|  |  |  |  |  |  | 10 | 0.67 | 0.85 | 6 | 7 | 41 | 3 |
|  |  |  |  |  |  | 11 | 0.56 | 0.96 | 5 | 2 | 46 | 4 |
|  |  |  |  |  |  | 12 | 0.22 | 0.98 | 2 | 1 | 47 | 7 |
|  |  |  |  |  |  | 13 | 0.22 | 0.98 | 2 | 1 | 47 | 7 |
|  |  |  |  |  |  | 14 | 0.11 | 0.98 | 1 | 1 | 47 | 8 |
| *N* = number; TP = true positive; FP = false positive; TN = true negative; FN = false negative; MDD = major depressive disorder | | | | | | | | | | | | |

| 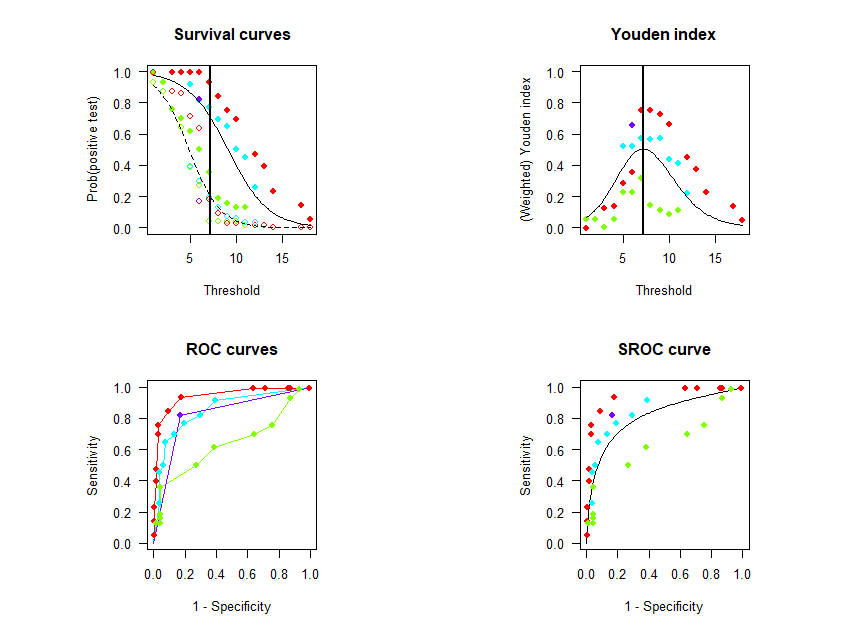 |
| --- |
| *Figure A6b.* Survival Curves, Youden Index, ROC and SROC Plots HADS-A |

| Table A6b.  *2x2 Table for the HADS-A* | | | | | | | | | | | | |
| --- | --- | --- | --- | --- | --- | --- | --- | --- | --- | --- | --- | --- |
|  |  | Reference standard | | | | Index test | | | 2x2 Table | | | |
| Study | Total, *N* | With diagnosis, *n* | Without diagnosis, *n* | Prevalence, % | Target Condition | Cut-off score | Sensitivity | Specificity | TP | FP | TN | FN |
| El-Rufaie et al. (1995) | 217 | 40 | 177 | 18.4 | Anxiety | 4/5 | 0.93 | 0.61 | 37 | 69 | 108 | 3 |
|  |  |  |  |  |  | 5/6 | 0.83 | 0.71 | 33 | 52 | 125 | 7 |
|  |  |  |  |  |  | 6/7 | 0.78 | 0.81 | 31 | 34 | 143 | 9 |
|  |  |  |  |  |  | 7/8 | 0.71 | 0.87 | 28 | 23 | 154 | 12 |
|  |  |  |  |  |  | 8/9 | 0.66 | 0.93 | 26 | 13 | 164 | 14 |
|  |  |  |  |  |  | 9/10 | 0.49 | 0.94 | 20 | 10 | 167 | 20 |
|  |  |  |  |  |  | 10/11 | 0.44 | 0.97 | 18 | 6 | 171 | 22 |
|  |  |  |  |  |  | 11/12 | 0.24 | 0.97 | 10 | 6 | 171 | 30 |
| Al-Adawi et al. (2007) | 68 | 34 | 34 | 50.0 | Anxiety | 0/1 | 1 | 0.06 | 34 | 32 | 2 | 0 |
|  |  |  |  |  |  | 1/2 | 0.94 | 0.12 | 32 | 30 | 4 | 2 |
|  |  |  |  |  |  | 2/3 | 0.77 | 0.24 | 26 | 26 | 8 | 8 |
|  |  |  |  |  |  | 3/4 | 0.71 | 0.36 | 24 | 22 | 12 | 10 |
|  |  |  |  |  |  | 4/5 | 0.62 | 0.62 | 21 | 13 | 21 | 13 |
|  |  |  |  |  |  | 5/6 | 0.50 | 0.74 | 17 | 9 | 25 | 17 |
|  |  |  |  |  |  | 6/7 | 0.35 | 0.97 | 12 | 1 | 33 | 22 |
|  |  |  |  |  |  | 7/8 | 0.18 | 0.97 | 6 | 1 | 33 | 28 |
|  |  |  |  |  |  | 8/9 | 0.15 | 0.97 | 5 | 1 | 33 | 29 |
|  |  |  |  |  |  | 9/10 | 0.12 | 0.97 | 4 | 1 | 33 | 30 |
|  |  |  |  |  |  | 10/11 | 0.12 | 1 | 4 | 0 | 34 | 30 |
| Al-Asmi et al. (2012) | 146 | 66 | 80 | 45.2 | Anxiety | 0/1 | 1 | 0 | 66 | 80 | 0 | 0 |
|  |  |  |  |  |  | 1/2 | N/R | N/R | N/R | N/R | N/R | N/R |
|  |  |  |  |  |  | 2/3 | 1 | 0.13 | 66 | 70 | 10 | 0 |
|  |  |  |  |  |  | 3/4 | 1 | 0.14 | 66 | 69 | 11 | 0 |
|  |  |  |  |  |  | 4/5 | 1 | 0.29 | 66 | 57 | 23 | 0 |
|  |  |  |  |  |  | 5/6 | 1 | 0.36 | 66 | 51 | 29 | 0 |
|  |  |  |  |  |  | 6/7 | 0.94 | 0.83 | 62 | 14 | 66 | 4 |
|  |  |  |  |  |  | 7/8 | 0.85 | 0.91 | 56 | 7 | 73 | 10 |
|  |  |  |  |  |  | 8/9 | 0.76 | 0.98 | 50 | 2 | 78 | 16 |
|  |  |  |  |  |  | 9/10 | 0.70 | 0.98 | 46 | 2 | 78 | 20 |
|  |  |  |  |  |  | 10/11 | N/R | N/R | N/R | N/R | N/R | N/R |
|  |  |  |  |  |  | 11/12 | 0.47 | 0.99 | 31 | 1 | 79 | 35 |
|  |  |  |  |  |  | 12/13 | 0.39 | 0.99 | 26 | 1 | 79 | 40 |
|  |  |  |  |  |  | 13/14 | 0.23 | 1.00 | 15 | 0 | 80 | 51 |
|  |  |  |  |  |  | 14/15 | N/R | N/R | N/R | N/R | N/R | N/R |
|  |  |  |  |  |  | 15/16 | N/R | N/R | N/R | N/R | N/R | N/R |
|  |  |  |  |  |  | 16/17 | 0.14 | 1 | 9 | 0 | 80 | 57 |
|  |  |  |  |  |  | 17/18 | 0.05 | 1 | 3 | 0 | 80 | 63 |
| Karam et al. (2018) | 57 | 13 | 44 | 22.8 | Anxiety | 5/6 | 0.85 | 0.83 | 11 | 7 | 37 | 2 |
| *N* = number; TP = true positive; FP = false positive; TN = true negative; FN = false negative; MDD = major depressive disorder | | | | | | | | | | | | |

| *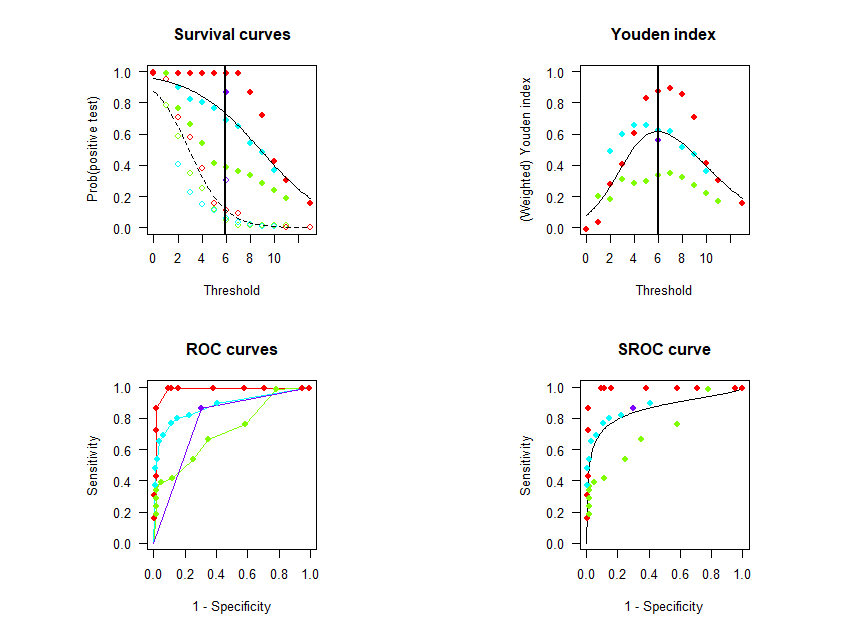* |
| --- |
| *Figure A6c.* Survival Curves, Youden Index, ROC and SROC Plots HADS-D |

| Table A6c.  *2x2 Table for the HADS-D* | | | | | | | | | | | | |
| --- | --- | --- | --- | --- | --- | --- | --- | --- | --- | --- | --- | --- |
|  |  | Reference standard | | | | Index test | | | 2x2 Table | | | |
| Study | Total, *N* | With diagnosis, *n* | Without diagnosis, *n* | Prevalence, % | Target Condition | Cut-off score | Sensitivity | Specificity | TP | FP | TN | FN |
| El-Rufaie et al. (1995) | 217 | 52 | 165 | 24.0 | Depression | 1/2 | 0.91 | 0.59 | 47 | 67 | 98 | 5 |
|  |  |  |  |  |  | 2/3 | 0.83 | 0.77 | 43 | 37 | 128 | 9 |
|  |  |  |  |  |  | 3/4 | 0.81 | 0.86 | 42 | 24 | 141 | 10 |
|  |  |  |  |  |  | 4/5 | 0.77 | 0.89 | 40 | 18 | 147 | 12 |
|  |  |  |  |  |  | 5/6 | 0.70 | 0.94 | 36 | 10 | 155 | 16 |
|  |  |  |  |  |  | 6/7 | 0.66 | 0.97 | 34 | 5 | 160 | 18 |
|  |  |  |  |  |  | 7/8 | 0.55 | 0.98 | 28 | 3 | 162 | 24 |
|  |  |  |  |  |  | 8/9 | 0.47 | 0.99 | 25 | 1 | 164 | 27 |
|  |  |  |  |  |  | 9/10 | 0.36 | 0.99 | 19 | 1 | 164 | 33 |
| Al-Adawi et al. (2007) | 68 | 39 | 29 | 57.4 | Depression | 0/1 | 1 | 0.21 | 39 | 23 | 6 | 0 |
|  |  |  |  |  |  | 1/2 | 0.77 | 0.41 | 30 | 17 | 12 | 9 |
|  |  |  |  |  |  | 2/3 | 0.67 | 0.66 | 26 | 10 | 19 | 13 |
|  |  |  |  |  |  | ¾ | 0.54 | 0.76 | 21 | 7 | 22 | 18 |
|  |  |  |  |  |  | 4/5 | 0.41 | 0.90 | 16 | 3 | 26 | 23 |
|  |  |  |  |  |  | 5/6 | 0.39 | 0.97 | 15 | 1 | 28 | 24 |
|  |  |  |  |  |  | 6/7 | 0.36 | 1 | 14 | 0 | 29 | 25 |
|  |  |  |  |  |  | 7/8 | 0.33 | 1 | 13 | 0 | 29 | 26 |
|  |  |  |  |  |  | 8/9 | 0.28 | 1 | 11 | 0 | 29 | 28 |
|  |  |  |  |  |  | 9/10 | 0.23 | 1 | 9 | 0 | 29 | 30 |
|  |  |  |  |  |  | 10/11 | 0.18 | 1 | 7 | 0 | 29 | 32 |
| Al-Asmi et al. (2012) | 150 | 40 | 110 | 36.4 | MDD | 0 | 1 | 0.00 | 40 | 110 | 0 | 0 |
|  |  |  |  |  |  | 0/1 | 1 | 0.05 | 40 | 105 | 5 | 0 |
|  |  |  |  |  |  | 0/2 | 1 | 0.29 | 40 | 78 | 32 | 0 |
|  |  |  |  |  |  | 2/3 | 1 | 0.42 | 40 | 64 | 46 | 0 |
|  |  |  |  |  |  | 3/4 | 1 | 0.62 | 40 | 42 | 68 | 0 |
|  |  |  |  |  |  | 4/5 | 1 | 0.85 | 40 | 17 | 93 | 0 |
|  |  |  |  |  |  | 5/6 | 1 | 0.89 | 40 | 12 | 98 | 0 |
|  |  |  |  |  |  | 6/7 | 1 | 0.91 | 40 | 10 | 100 | 0 |
|  |  |  |  |  |  | 7/8 | 0.88 | 0.99 | 35 | 1 | 109 | 5 |
|  |  |  |  |  |  | 8/9 | 0.73 | 0.99 | 29 | 1 | 109 | 11 |
|  |  |  |  |  |  | 9/10 | 0.43 | 0.99 | 17 | 1 | 109 | 23 |
|  |  |  |  |  |  | 10/11 | 0.30 | 1 | 12 | 0 | 110 | 28 |
|  |  |  |  |  |  | 11/12 | N/R | N/R | N/R | N/R | N/R | N/R |
|  |  |  |  |  |  | 1213 | 0.15 | 1 | 6 | 0 | 110 | 34 |
| Karam et al. (2018) | 57 | 10 | 47 | 17.5 | Depression | 5/6 | 0.90 | 0.70 | 9 | 14 | 33 | 1 |
| *N* = number; TP = true positive; FP = false positive; TN = true negative; FN = false negative; MDD = major depressive disorder | | | | | | | | | | | | |

| *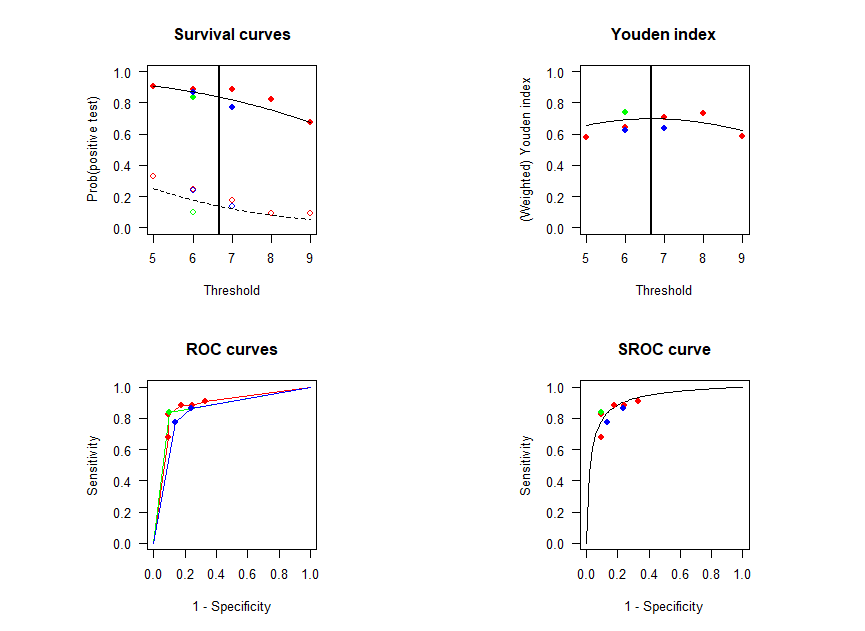* |
| --- |
| *Figure A6d.* Survival Curves, Youden Index, ROC and SROC Plots GDS-15 |

| Table A6d.  *2x2 Table for the GDS-15* | | | | | | | | | | | | |
| --- | --- | --- | --- | --- | --- | --- | --- | --- | --- | --- | --- | --- |
|  |  | Reference standard | | | | Index test | | | 2x2 Table | | | |
| Study | Total, *N* | With diagnosis, *n* | Without diagnosis, *n* | Prevalence, % | Target Condition | Cut-off score | Sensitivity | Specificity | TP | FP | TN | FN |
| Chaaya et al. (2008) | 105 | 47 | 58 | 44.8 | MDD/dysthymia | 5 | 0.92 | 0.67 | 43 | 19 | 39 | 4 |
|  |  |  |  |  |  | 6 | 0.89 | 0.76 | 42 | 14 | 44 | 5 |
|  |  |  |  |  |  | 7 | 0.89 | 0.83 | 42 | 10 | 48 | 5 |
|  |  |  |  |  |  | 8 | 0.83 | 0.91 | 39 | 5 | 53 | 8 |
|  |  |  |  |  |  | 9 | 0.68 | 0.91 | 32 | 5 | 53 | 15 |
| Hashim (2018) | 279 | 173 | 106 | 62.0 | MDD | 6 | 0.84 | 0.91 | 145 | 10 | 96 | 28 |
| Karam et al. (2018) | 57 | 10 | 47 | 17.5 | MDD | 6 | 0.90 | 0.76 | 9 | 11 | 36 | 1 |
|  |  |  |  |  |  | 7 | 0.80 | 0.87 | 8 | 6 | 41 | 2 |
| *N* = number; TP = true positive; FP = false positive; TN = true negative; FN = false negative; MDD = major depressive disorder | | | | | | | | | | | | |

| Table A6e.  *Summary Operating Points of Sensitivity and Specificity for the GDS-15* | | | | | |
| --- | --- | --- | --- | --- | --- |
| Cut-off | Studies *n* | Participants *n* | Sensitivity %  (95% CI) | Specificity %  (95% CI) | Pooled AUC  (95% CI)* |
| 5 | 1 | 105 | 90.7 (84.7-94.6) | 74.8 (57.4-86.8) | .916 (.869-.948) |
| 6 | 3 | 341 | 87.0 (79.4-92.0) | 82.2 (68.0-91.0) |  |
| **7^b^** | **2** | **162** | **81.9 (72.6-88.6)** | **87.8 (76.7-94.0)** |  |
| 8 | 1 | 105 | 75.5 (63.9-84.2) | 91.8 (83.4-96.1) |  |
| 9 | 1 | 105 | 67.6 (53.8-79.0) | 94.5 (88.3-97.6) |  |
| *We reported the 95% CI of the AUC for sensitivity given specificity; ^a^ The model estimated an optimal threshold for the GDS-15 of 6.67 (sensitivity = 83.7 and specificity = 86.1). | | | | | |

| *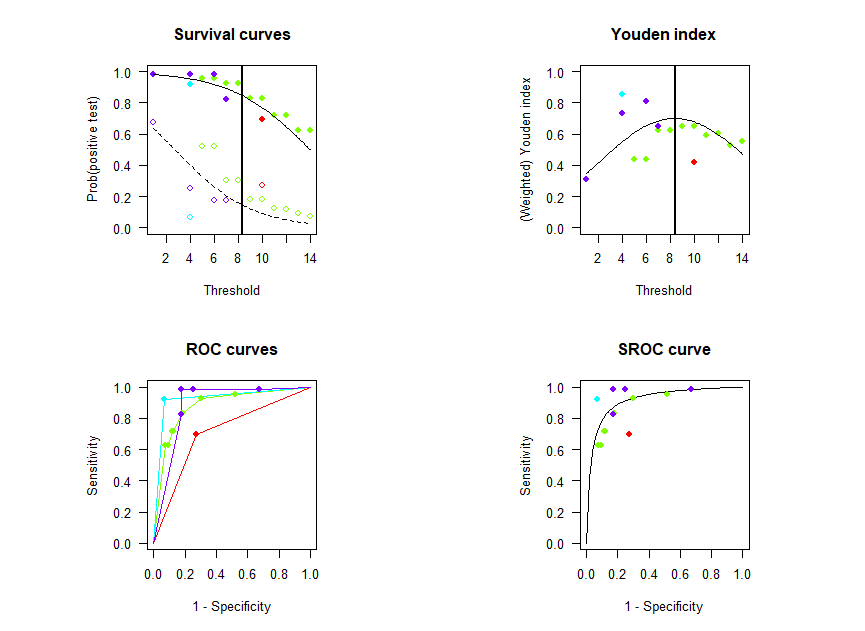* |
| --- |
| *Figure A6e.* Survival Curves, Youden Index, ROC and SROC Plots SRQ-20 |

| Table A6f.  *2x2 Table for the SRQ-20* | | | | | | | | | | | | |
| --- | --- | --- | --- | --- | --- | --- | --- | --- | --- | --- | --- | --- |
|  |  | Reference standard | | | | Index test | | | 2x2 Table | | | |
| Study* | Total, *N* | With diagnosis, *n* | Without diagnosis, *n* | Prevalence, % | Target Condition | Cut-off score | Sensitivity | Specificity | TP | FP | TN | FN |
| Climent et al. (1989) | 63 | N/R | N/R | N/R | CMD | 3/4 | 0.93 | 0.95 | 39 | 1 | 20 | 3 |
| El-Rufaie et al. (1994) | 217 | N/R | N/R | N/R | CMD | 5/6 | 0.78 | 0.75 | N/R | N/R | N/R | N/R |
| Al-Subaie et al. (1998) | 292 | 142 | 150 | 48.6 | CMD | 4/5 | 0.96 | 0.48 | 136 | 78 | 72 | 6 |
|  |  |  |  |  |  | 5/6 | 0.96 | 0.48 | 136 | 78 | 72 | 6 |
|  |  |  |  |  |  | 6/7 | 0.93 | 0.70 | 132 | 45 | 105 | 10 |
|  |  |  |  |  |  | 7/8 | 0.93 | 0.70 | 132 | 45 | 105 | 10 |
|  |  |  |  |  |  | 8/9 | 0.83 | 0.82 | 118 | 27 | 123 | 24 |
|  |  |  |  |  |  | 9/10 | 0.83 | 0.82 | 118 | 27 | 123 | 24 |
|  |  |  |  |  |  | 10/11 | 0.72 | 0.88 | 102 | 18 | 132 | 40 |
|  |  |  |  |  |  | 11/12 | 0.72 | 0.89 | 102 | 17 | 134 | 40 |
|  |  |  |  |  |  | 12/13 | 0.63 | 0.91 | 89 | 14 | 137 | 53 |
|  |  |  |  |  |  | 13/14 | 0.63 | 0.93 | 89 | 11 | 140 | 53 |
| Al-Arabi et al. (1999) | 49 | 17 | 30 | 35.0 | CMD | 9/10 | 0.71 | 0.72 | 12 | 8 | 22 | 5 |
| Llosa et al. (2017) | 55 | 30 | 25 | 45.5 | CMD | 0/1 | 1 | 0.33 | 30 | 17 | 8 | 0 |
|  |  |  |  |  |  | 1/2 | N/R | N/R | N/R | N/R | N/R | N/R |
|  |  |  |  |  |  | 2/3 | N/R | N/R | N/R | N/R | N/R | N/R |
|  |  |  |  |  |  | 3/4 | 1 | 0.77 | 30 | 6 | 19 | 0 |
|  |  |  |  |  |  | 4/5 | N/R | N/R | N/R | N/R | N/R | N/R |
|  |  |  |  |  |  | 5/6 | 1 | 0.83 | 30 | 4 | 21 | 0 |
|  |  |  |  |  |  | 6/7 | 0.83 | 0.86 | 25 | 4 | 21 | 5 |
| *N* = number; TP = true positive; FP = false positive; TN = true negative; FN = false negative; CMD = common mental disorder; *Alsuwaida et al (2006) was excluded from the meta-analysis because they also included 1 item enquiring about psychosis, while the other studies only used the 20 non-psychotic items. | | | | | | | | | | | | |
