# Supplementary material for: A systematic review and meta-analysis of diagnostic test accuracy studies of self-report screening instruments for common mental disorders in Arabic-speaking adults
Source: Glob Ment Health (Camb). 2021 Nov 23;8:e43. doi: 10.1017/gmh.2021.39 (PMC8679833; doi:10.1017/gmh.2021.39)
Supplement: Supplementary file 1 [file S205442512100039Xsup001.zip › Appendix 5. Figures ROC.docx]

**Supplementary material**

**Appendix 5**

**Figures for instruments with at least 3 studies**

| 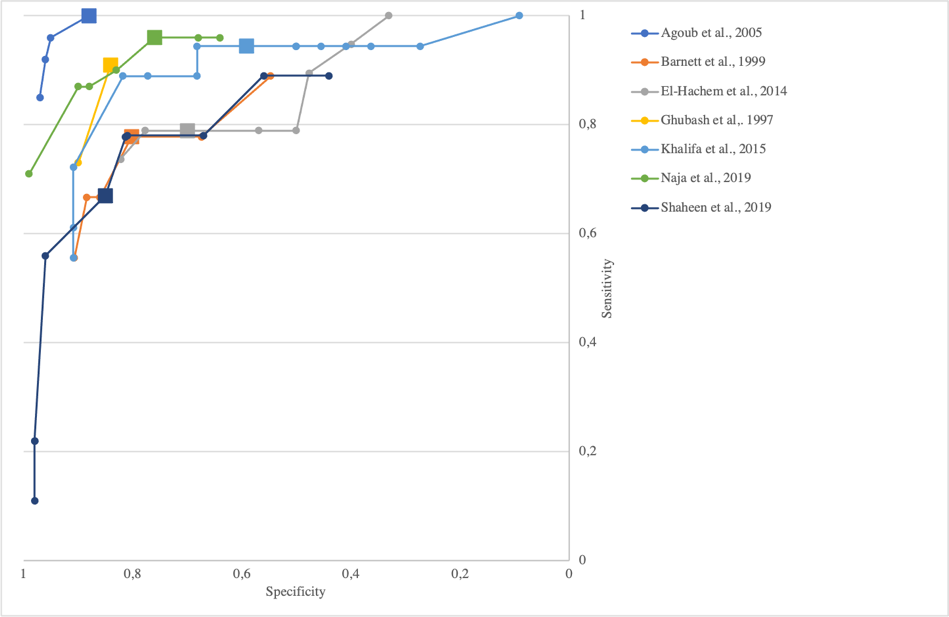   = cut-off 9/10 | **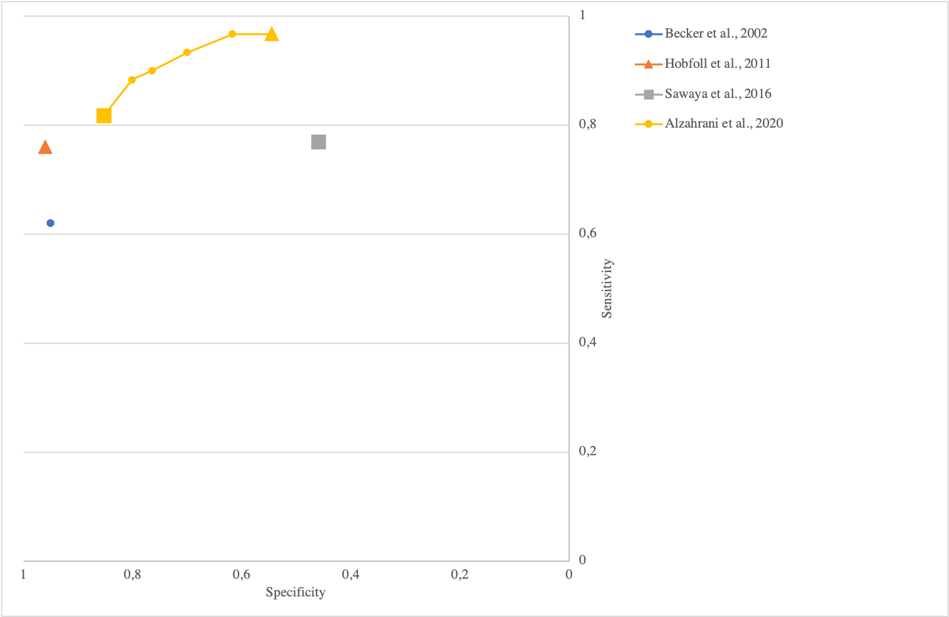**   = cut-off 9/10  ∆ = cut-off 4/5 |
| --- | --- |
| *Figure 2a.* ROC Curves EPDS | *Figure 2b.* ROC Curves PHQ-9 |
| 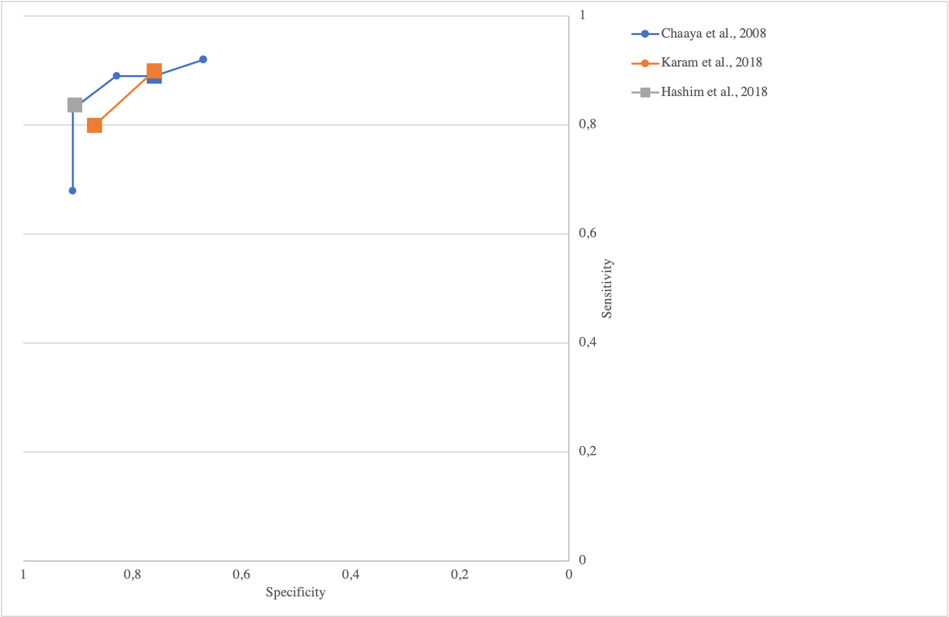   = cut-off 5/6 | 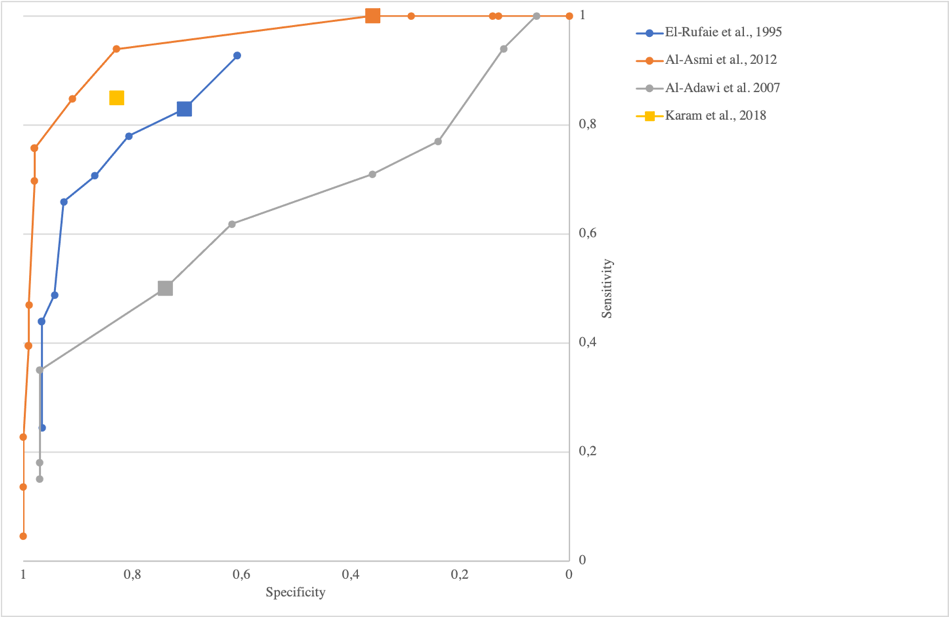   = cut-off 5/6 |
| *Figure 2c.* ROC Curves GDS-15 | *Figure 2d.* ROC Curves HADS-A |
| 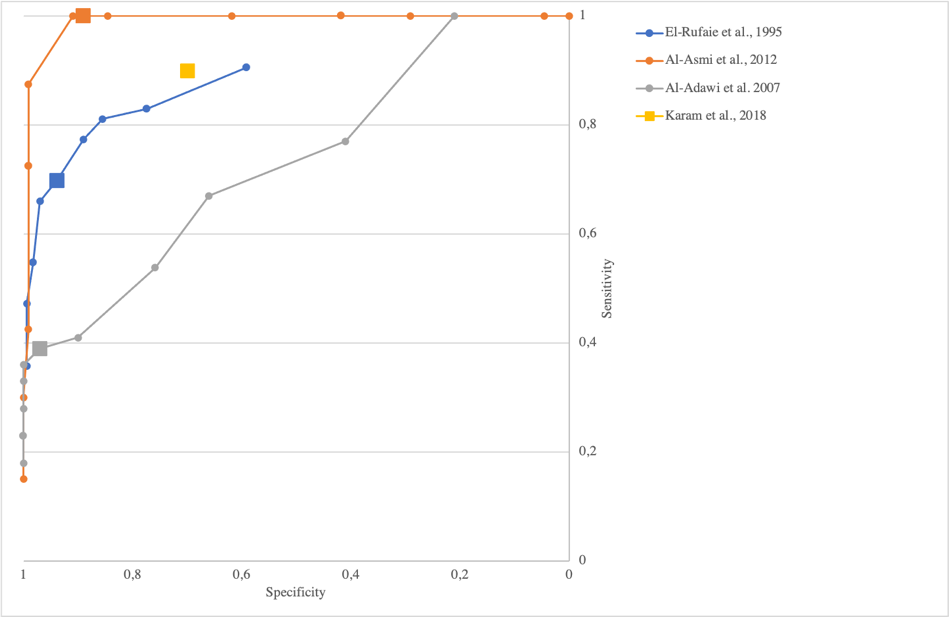   = cut-off 5/6 | 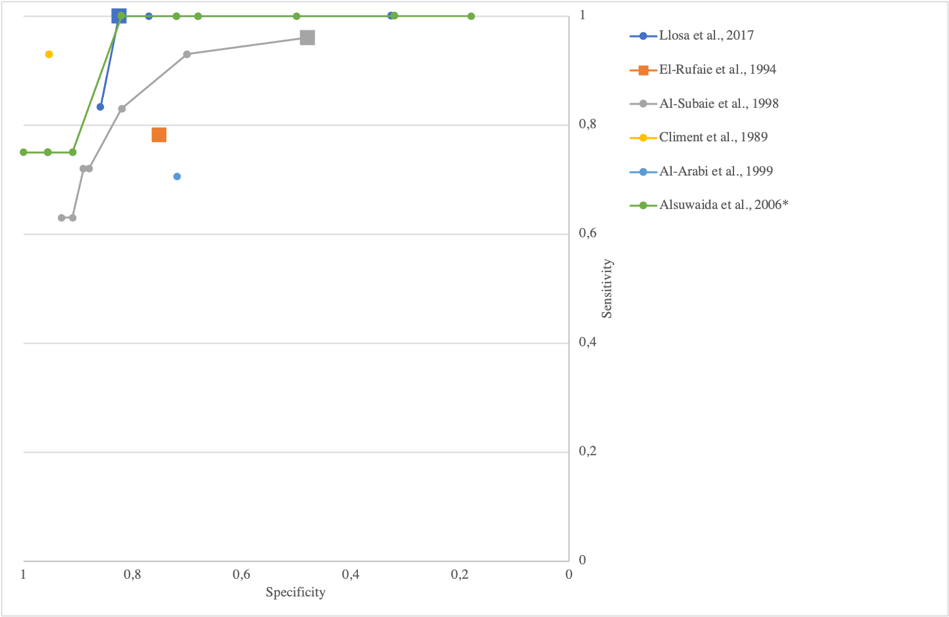   = cut-off 5/6 |
| *Figure 2e.* ROC Curves HADS-D | *Figure 2f.* ROC Curves SRQ-20 |
| EPDS = Edinburgh Postnatal Depression Scale; PHQ-9 = Patient Health Questionnaire; GDS-15 = Geriatric Depression Scale; HADS-A = Hospital Anxiety and Depression Scale, anxiety subscale; HADS-D = Hospital Anxiety and Depression Scale, depression subscale; SRQ-20 = 20-item Self Reporting Questionnaire; ROC = receiver operating characteristic; * Alsuwaida et al. (2006) used 21 items of the SRQ, while the other studies used the 20 non-psychotic items only. | |
