# Supplementary material for: A systematic review and meta-analysis of diagnostic test accuracy studies of self-report screening instruments for common mental disorders in Arabic-speaking adults
Source: Glob Ment Health (Camb). 2021 Nov 23;8:e43. doi: 10.1017/gmh.2021.39 (PMC8679833; doi:10.1017/gmh.2021.39)
Supplement: Supplementary file 1 [file S205442512100039Xsup001.zip › Appendix 4. Description of index tests.docx]

**Supplementary material**

**Appendix 1**

| Table 1.  *Overview Index Tests* | |  | | |  |
| --- | --- | --- | --- | --- | --- |
| **Index test** | **Studies in review** | **Scale description/construct** | **# items** | **Response options/scale** | **Reference time** |
| **Screening instruments for depression** | | |  |  |  |
| EPDS | (Agoub et al., 2005; Barnett et al., 1999; El-Hachem et al., 2014; Ghubash et al., 1997; Khalifa et al., 2015; Naja et al., 2019; Shaheen et al., 2019) | The EPDS screens for risk factors for the development of postnatal depression in community samples. The scale does not provide a measure of severity of the illness, but is meant as a predictor of the probability of postpartum depression. | 10 | 4-point Likert scale (0-3); sum score range 0-30. High score indicates worse symptomatology. | Past week |
| PHQ-9 | (Alzahrani et al., 2020; Becker et al., 2002; Hobfoll et al., 2011; Sawaya et al., 2016) | The PHQ-9 is a self-report questionnaire used to measure symptoms of major depressive disorder (DSM-IV). | 9 | Severity score: 4-point Likert scale (0-3); sum score range 0-27. High score indicates worse symptomatology.  Provisional diagnosis: MDD if item ‘low mood’ or ‘loss of interest’ are scored >1 and in total at least 5 items of the scale are scored >1 (suicidality >0); Other depressive syndrome if item ‘low mood’ or ‘loss of interest’ are scored >1 and in total 2 to 4 items of the scale are scored >1 (suicidality >0). Other disorders should be ruled out. | Past 2 weeks |
| GDS-15 | (Chaaya et al., 2008; Hashim, 2018; Karam et al., 2018) | The GDS-15 is a self-rating scale to assess depression in non-demented older adults. | 15 | Yes (1) / no (0); sum score range 0-15. High score indicates worse symptomatology. | Past week |
| BDI-II | (Naja et al., 2019) | The BDI-II is screens for the presence and severity of symptoms of major depressive disorder according to the DSM-IV. | 21 | 4-point Likert scale (0-3); sum score range 0-6. High score indicates worse symptomatology. | Past 2 weeks |
| CES-D | (Ghubash et al., 2000) | The CES-D measures the level of depressive symptomatology. | 20 | 4-point Likert scale (0-3); sum score range 0-60. High score indicates worse symptomatology. | Past week |
| MDI | (Fawzi et al., 2012) | The MDI is a based on the DSM-IV symptoms of major depression and the ICD-10 category of moderate to severe depression. | 12 | 6-point Likert scale ranging (0-5); sum score range 0-50. High score indicates worse symptomatology. | Past 2 weeks |
| AES | (Al-Adawi et al., 2004) | The AES has been developed to measure severity of apathy. | 18 | 4-point Likert scale (1-4); sum score range 18-72. High score indicates increased apathic state. | Past 4 weeks |
| WHO-5 | (Sibai et al., 2009) | The WHO-5 is a brief questionnaire originally developed by the WHO to measure emotional well-being among diabetic patients. | 5 | 6-point Likert scale (0-5); sum score range 0-25. Higher score indicates higher level of reported wellbeing. | Past two weeks |
| PSST | (Mahfoud et al., 2019) | This questionnaire was developed for use by clinicians to screen for premenstrual dysphoric disorder. | 19 | 4-point Likert scale ranging from *not at all* to *severe*. The following criteria must be present for a diagnosis of PMDD: 1) at least one of #1-4 is *severe*, 2) at least four of #1-14 are *moderate/severe*, and 3) at least one of #A-E is *severe*. | Refers to symptoms that start a few days before, and stop a few days after a woman’s period |
| **Screening instruments for anxiety** | | |  |  |  |
| GAD-7 | (Sawaya et al., 2016) | The GAD-7 is a self-report scale based on DSM-IV criteria and used to measure the severity of (generalized) anxiety disorder. | 7 | 4-point Likert scale (0-3); sum score range 0-21. High score indicates worse symptomatology. | Past 2 weeks |
| Anxiety modules PHQ (Panic and GAD) | (Becker et al., 2002) | The panic and generalized anxiety disorder (GAD) modules are part of the larger Patient Health Questionnaire (PHQ). The PHQ is a self-report questionnaire to measure symptoms of depression, anxiety, somatoform, alcohol and eating disorders. | 15 (panic) + 7 (GAD) | Severity score: Yes (1) / No (0);  Provisional diagnosis: Panic syndrome if panic items 3a-d are all scored Yes and >3 of 4a-k are scored Yes; Other anxiety syndrome if item 5a and answers to three or more of items 5b-9 are scored more than of the days. Other disorders should be ruled out. | Past 4 weeks |
| **Screening instruments for anxiety and depression** | | |  |  |  |
| HADS | (Al-Adawi et al., 2007; Al-Asmi et al., 2012; El-Rufaie & Absood, 1995; Karam et al., 2018) | The HADS is a self-report questionnaire used to measure symptoms associated with anxiety and depression. The scale consists of separate scales for anxiety (7 items) and depression (7 items). | 14 | 4-point Likert scale (0-3); sum score range (anxiety subscale) 0-21; sum score range (depression subscale) 0-2. High scores indicate worse symptomatology. | Past week |
| HSCL-25 | (Mahfoud et al., 2013) | The HSCL-25 is a questionnaire composed of two subscales on anxiety (10 items) and depression (15 items). | 25 | 4-point Likert scale (1-4); mean of (subscale) items used as total score (1-4). High scores indicate worse symptomatology. | Past week/month |
| PCAD | (El-Rufaie et al., 1997) | The PCAD scale is composed of depression, anxiety and somatic items from the HADS and SRQ-20 scales. | 12 | Unclear | N/R |
|  |  | **PTSD instruments** |  |  |  |
| SPTSS | (Caspi et al., 2007) | The SPTSS screens for PTSD symptoms not specifically linked to a particular traumatic event. | 10 | 11-point Likert-scale (0-10); sum score range 0-100. High score indicates worse symptomatology. | Past two weeks |
| **Screening instruments for psychological distress** | | |  |  |  |
| SRQ-20 | (Al-Arabi et al., 1999; Al-Subaie et al., 1998; Climent et al., 1989; El-Rufaie & Absood, 1994; Llosa et al., 2017) | The SRQ-20 measures non-psychotic symptoms of common mental disorders developed by the WHO to detect CMDs. | 20 | Yes (1) / No (0); sum score range 0-20. High score indicates worse symptomatology. | Past month |
| GHQ-12 | (El-Rufaie & Daradkeh, 1996) | The GHQ-12 is a screening tool to identify potential non-psychotic psychiatric cases. | 12 | 4-point Likert scale (0-3); sum score range 0-36 or 0-12 for bi-modal scoring (0-0-1-1). High score indicates worse symptomatology. | Past few weeks |
