# Supplementary material for: A systematic review and meta-analysis of diagnostic test accuracy studies of self-report screening instruments for common mental disorders in Arabic-speaking adults
Source: Glob Ment Health (Camb). 2021 Nov 23;8:e43. doi: 10.1017/gmh.2021.39 (PMC8679833; doi:10.1017/gmh.2021.39)
Supplement: Supplementary file 1 [file S205442512100039Xsup001.zip › Appendix 3. Risk of bias description.docx]

**Supplementary material**

**Appendix 2**

**Risk of bias assessment - Signaling questions with interpretations**

The following signaling questions are used to assess the risk of bias according to the QUADAS-2 (Whiting et al., 2011). QUADAS-2 is a generic set of criteria consisting of four key domains, i.e., patient selection, index test, reference standard and flow of patients through the study and timing of the index test and reference standard. If the answer on a question is Yes, the risk of bias is low.

We added two items to account for biases specific to the use of (semi-)structured psychiatric interviews. These extra items concerned 1) whether studies used a semi-structured interview (Yes) or clinician diagnosis (No) (domain 3), 2) whether data on interviewer variation (i.e., inter-rater reliability or agreement) for the (semi)structured interview fell within an acceptable range (domain 3), and 3) whether all participants received a reference standard (domain 4).

**Domain 1 – Patient selection**

1.1 Was a consecutive or random sample of patients enrolled?

*We answered “Yes” if a consecutive or random sample was described, and “Unclear” if no mention was made of the sampling method. However, if unclear, we assumed this did not lead to a high risk of bias on this domain.*

1.2 Was a case-control design avoided?

*We only considered designs to be case-control if this was based on the reference standard or based on selection of participants (e.g., patients with depressive disorder versus healthy controls), but not if based on the index test. We did not consider other medical (non-psychiatric) conditions (e.g., sample including participants with and without medical condition) for this item.*

1.3 Did the study avoid inappropriate exclusions?

*We answered “Unclear” if no mention was made of exclusion criteria. However, this indication would not automatically lead to an unclear risk of bias for this domain (as we assumed that the absence of reporting of exclusion criteria would mean there were no exclusion criteria and thus no inappropriate exclusions). History of psychological illness was considered an inappropriate exclusion criterion.*

**Domain 2 – Index test**

2.1 Were the index test results interpreted without knowledge of the results of the reference standard?

*We answered “Yes” if the index test was completed before conducting the reference standard or when administrators were blinded.*

2.2 If a threshold was used, was it pre-specified?

*We answered “Yes” if the cut-off score was pre-specified. Selection of a cut-off that optimized the diagnostic accuracy may lead to overoptimistic estimates of test performance (Whiting et al., 2011).*

**Domain 3 – Reference standard**

3.1 Is the reference standard likely to correctly classify the target condition?
*We included studies using either a (semi-)structured clinical interview, such as the Structured Clinical Interview for DSM (SCID), or a clinician diagnosis based on DSM or ICD criteria. Both were considered to correctly classify the target condition of any common mental disorder.*

3.2 Additional signaling question: Was a semi-structured clinical interview used (i.e., no clinician diagnosis)?

*We added this item to flag studies using a clinician diagnosis instead of a (semi-)structured interview, because clinician interviews are unstructured and can therefore be considered to be less valid compared to semi- or fully structured interviews* (Mueller & Segal, 2015)*.*

3.3 Were the reference standard results interpreted without knowledge of the results of the index test?

*We answered “Unclear” if no mention of blinding was made.*

3.4 Additional signaling question: Were data on interviewer variation (i.e., inter-rater reliability or agreement) for the (semi-) structured interview within an acceptable range?

We answered “Yes” to this question if inter-rater reliability/agreement was perceived adequate (e.g., Cohen’s Kappa >.80) (McHugh, 2012), we answered “N/A” in case of only one interviewer, and we answered “Unclear” in case of any unclarity regarding the number of interviewers and/or the absence of reporting on the inter-rater reliability/agreement.

**Domain 4 – Flow and Timing**

4.1 Was there an appropriate interval between index test and reference standard?

*We answered “Yes” if the time interval was maximally 7 days. For those studies with a time interval of >7 days, we took the duration into account (e.g., one month versus 14 days) when assessing risk of bias.*

4.2 Additional signaling question: Did all patients receive a reference standard?

*We answered “No” in case of drop-out or sub-sample selected to undergo the reference standard.*

4.3 Did all patients receive the same reference standard?

*We answered “Yes” if all participants (from the selected random sub-sample) underwent the same clinical interview.*

4.4 Were all patients included in the analysis?

*We answered “Yes” in case all eligible participants were included in the analysis, but also if all participants from a random sub-sample were included in the analysis. We answered “No” in case of drop-out >20%.*

**Literature**

McHugh, M. L. (2012). Lessons in biostatistics interrater reliability : the kappa statistic. *Biochemica Medica*, *22*(3), 276–282. https://hrcak.srce.hr/89395

Mueller, A. E., & Segal, D. L. (2015). Structured versus Semistructured versus Unstructured Interviews. In R. L. Cautin & S. O. Lilienfeld (Eds.), *The Encyclopedia of Clinical Psychology* (1st ed., Issue August, pp. 1–7). John Wiley & Sons, Inc. https://doi.org/10.1002/9781118625392.wbecp069

Whiting, P. F., Rutjes, A. W. S., Westwood, M. E., Mallett, S., Deeks, J. J., Reitsma, J. B., Leeflang, M. M. G., Sterne, J. A. C., Bossuyt, P. M. M., & Group, the Q.-2. (2011). QUADAS-2: A Revised Tool for the Quality Assessment of Diagnostic Accuracy Studies. *Ann Intern Med*, *155*(8), 529–536.
