# Supplementary material for: A systematic review and meta-analysis of diagnostic test accuracy studies of self-report screening instruments for common mental disorders in Arabic-speaking adults
Source: Glob Ment Health (Camb). 2021 Nov 23;8:e43. doi: 10.1017/gmh.2021.39 (PMC8679833; doi:10.1017/gmh.2021.39)
Supplement: Supplementary file 1 [file S205442512100039Xsup001.zip › Appendix 2. Search strategy.docx]

**Supplementary material**

**Appendix 1**

**Full search strategy**

**PubMed Session Results (22 Jan 2021)**

| **#** | **Query** | **Results** |
| --- | --- | --- |
| #6 | #1 AND #2 AND #3 AND #4 AND #5 | 1,150 |
| #5 | "Middle East"[Mesh] OR Middle East*[tw] OR Algeria*[tw] OR Bahrain*[tw] OR Chad*[tw] OR Comoros[tw] OR Djibouti*[tw] OR Egypt*[tw] OR Eritrea*[tw] OR Iraq*[tw] OR Israel*[tw] OR Jordan*[tw] OR Kuwait*[tw] OR Leban*[tw] OR Libya*[tw] OR Malta[tw] OR Maltes*[tw] OR Mauritania*[tw] OR Morocc*[tw] OR Oman*[tw] OR Palestin*[tw] OR Qatar*[tw] OR Saudi*[tw] OR Somalia*[tw] OR Sudan*[tw] OR Syria*[tw] OR Tanzan*[tw] OR Tunis*[tw] OR UAE[tw] OR Yemen*[tw] OR Cyprus[tw] OR Cypriot*[tw] OR Iran*[tw] OR Mali[tw] OR Niger[tw] OR Senegal*[tw] OR Turkey[tw] OR Turks[tw] OR arab[tw] OR arabic[tw] | 367,508 |
| #4 | "Sensitivity and Specificity"[Mesh] OR Sensitivity[tiab] OR Specificity[tiab] OR ROC[tiab] OR "Receiver Operating Characteristic*"[tiab] OR "Receiver Operative Characteristic*"[tiab] OR "Receiver Operating Curve*"[tiab] OR "Receiver Operative Curve*"[tiab] OR accuracy[tiab] OR validit*[tiab] OR validat*[tiab] OR "Detection Limit*"[tiab] OR "Limits of Detection"[tiab] OR "Limit of Detection"[tiab] OR "Predictive Value of Test*"[tiab] OR "false positive"[tiab] OR "false negative"[tiab] OR likelyhood*[tiab] OR likelihood*[tiab] OR pretest[tiab] OR "pre-test"[tiab] OR posttest[tiab] OR "post-test"[tiab] OR "Reproducibility of results"[Mesh] | 2,686,753 |
| #3 | "Reference Standards"[Mesh] OR "Interview, Psychological"[Mesh] OR "reference standard*"[tiab] OR "reference test*"[tiab] OR "gold standard*"[tiab] OR "golden standard*"[tiab] OR clinical*[tiab] OR clinician*[tiab] OR diagnos*[tiab] OR neuropsychiatric*[tiab] OR "neuro-psychiatric*"[tiab] OR interview*[tiab] OR examination*[tiab] OR schedule*[tiab] OR structured[tiab] OR "semi-structured"[tiab] OR semistructured[tiab] OR "symptom scale*"[tiab] OR MINI[tiab] OR CDI[tiab] OR CIDI[tiab] OR CIS[tiab] OR SCID[tiab] | 6,793,602 |
| #2 | "Diagnostic Self Evaluation"[Mesh] OR "self-evaluation*"[tiab] OR "self-report*"[tiab] OR selfreport*[tiab] OR "self-assessment*"[tiab] OR "self-disclosure*"[tiab] OR "Surveys and Questionnaires"[Mesh:NoExp] OR "Patient Reported Outcome Measures"[Mesh] OR "Self Report"[Mesh] OR questionnaire*[tiab] OR "Psychiatric Status Rating Scales"[Mesh] OR "Psychological Tests"[Mesh:NoExp] OR "Self-Assessment"[Mesh] OR "Self Disclosure"[Mesh] OR screening[tiab] OR screener*[tiab] OR index[tiab] OR indices[tiab] OR instrument[tiab] OR instruments[tiab] OR measure[tiab] OR measures[tiab] OR scale[tiab] OR scales[tiab] OR scaling[tiab] OR survey[tiab] OR surveys[tiab] OR tool*[tiab] OR test[tiab] OR tests[tiab] OR checklist*[tiab] OR inventor*[tiab] | 6,100,694 |
| #1 | "Mental Disorders"[Mesh:NoExp] OR "Stress, Psychological"[Mesh] OR "Mood Disorders"[Mesh] OR "Anxiety Disorders"[Mesh] OR "Diagnostic and Statistical Manual of Mental Disorders"[Mesh] OR "Anxiety"[Mesh:NoExp] OR "Depression"[Mesh] OR "psychological distress"[tiab] OR "psychological stress"[tiab] OR anxiety[tiab] OR anxieties[tiab] OR anxious[tiab] OR depress*[tiab] OR phobi*[tiab] OR panic[tiab] OR "common mental disorder*"[tiab] OR "Stress Disorders, Traumatic"[Mesh:NoExp] OR "Stress Disorders, Post-Traumatic"[Mesh] OR "Stress Disorders, Traumatic, Acute"[Mesh] OR "Acute Stress Disorder*"[tiab] OR "Post-Traumatic Stress Disorder*"[tiab] OR "Posttraumatic Stress Disorder*"[tiab] OR PTSD[tiab] | 959,631 |

**Embase.com Session Results (22 Jan 2021)**

| **#** | **Query** | **Results** |
| --- | --- | --- |
| #6 | #1 AND #2 AND #3 AND #4 AND #5 | 1,532 |
| #5 | 'Middle East'/exp OR 'Middle East':ab,ti,kw OR Algeria*:ab,ti,kw OR Bahrain*:ab,ti,kw OR Chad*:ab,ti,kw OR Comoros:ab,ti,kw OR Djibouti*:ab,ti,kw OR Egypt*:ab,ti,kw OR Eritrea*:ab,ti,kw OR Iraq*:ab,ti,kw OR Israel*:ab,ti,kw OR Jordan*:ab,ti,kw OR Kuwait*:ab,ti,kw OR Leban*:ab,ti,kw OR Libya*:ab,ti,kw OR Malta:ab,ti,kw OR Maltes*:ab,ti,kw OR Mauritania*:ab,ti,kw OR Morocc*:ab,ti,kw OR Oman*:ab,ti,kw OR Palestin*:ab,ti,kw OR Qatar*:ab,ti,kw OR Saudi*:ab,ti,kw OR Somalia*:ab,ti,kw OR Sudan*:ab,ti,kw OR Syria*:ab,ti,kw OR Tanzan*:ab,ti,kw OR Tunis*:ab,ti,kw OR UAE:ab,ti,kw OR Yemen*:ab,ti,kw OR Cyprus:ab,ti,kw OR Cypriot*:ab,ti,kw OR Iran*:ab,ti,kw OR Mali:ab,ti,kw OR Niger:ab,ti,kw OR Senegal*:ab,ti,kw OR Turkey:ab,ti,kw OR Turks:ab,ti,kw OR arab:ab,ti,kw OR arabic:ab,ti,kw | 449,589 |
| #4 | 'sensitivity and specificity'/exp OR Sensitivity:ab,ti,kw OR Specificity:ab,ti,kw OR ROC:ab,ti,kw OR 'Receiver Operating Characteristic*':ab,ti,kw OR 'Receiver Operative Characteristic*':ab,ti,kw OR 'Receiver Operating Curve*':ab,ti,kw OR 'Receiver Operative Curve*':ab,ti,kw OR accuracy:ab,ti,kw OR validit*:ab,ti,kw OR validat*:ab,ti,kw OR 'Detection Limit*':ab,ti,kw OR 'Limits of Detection':ab,ti,kw OR 'Limit of Detection':ab,ti,kw OR 'Predictive Value of Test*':ab,ti,kw OR 'false positive':ab,ti,kw OR 'false negative':ab,ti,kw OR likelyhood*:ab,ti,kw OR likelihood*:ab,ti,kw OR pretest:ab,ti,kw OR 'pre-test':ab,ti,kw OR posttest:ab,ti,kw OR 'post-test':ab,ti,kw OR 'reproducibility'/exp | 3,164,568 |
| #3 | 'standard'/de OR 'gold standard'/exp OR 'psychological interview'/exp OR 'reference standard*':ab,ti,kw OR 'reference test*':ab,ti,kw OR 'gold* standard*':ab,ti,kw OR clinical*:ab,ti,kw OR clinician*:ab,ti,kw OR diagnos*:ab,ti,kw OR neuropsychiatric*:ab,ti,kw OR 'neuro-psychiatric*':ab,ti,kw OR interview*:ab,ti,kw OR examination*:ab,ti,kw OR schedule*:ab,ti,kw OR structured:ab,ti,kw OR 'semi-structured':ab,ti,kw OR semistructured:ab,ti,kw OR 'symptom scale*':ab,ti,kw OR MINI:ab,ti,kw OR CDI:ab,ti,kw OR CIDI:ab,ti,kw OR CIS:ab,ti,kw OR SCID:ab,ti,kw | 9,705,302 |
| #2 | 'self evaluation'/exp OR 'Self-Evaluation*':ab,ti,kw OR 'self-report*':ab,ti,kw OR selfreport*:ab,ti,kw OR 'self-assessment*':ab,ti,kw OR 'self-disclosure*':ab,ti,kw OR 'questionnaire'/exp OR 'patient-reported outcome'/exp OR 'self report'/exp OR questionnaire*:ab,ti,kw OR 'psychological rating scale'/exp OR 'psychologic test'/de OR 'self disclosure'/exp OR screening:ab,ti,kw OR screener*:ab,ti,kw OR index:ab,ti,kw OR indices:ab,ti,kw OR instrument:ab,ti,kw OR instruments:ab,ti,kw OR measure:ab,ti,kw OR measures:ab,ti,kw OR scale:ab,ti,kw OR scales:ab,ti,kw OR scaling:ab,ti,kw OR survey:ab,ti,kw OR surveys:ab,ti,kw OR tool*:ab,ti,kw OR test:ab,ti,kw OR tests:ab,ti,kw OR checklist*:ab,ti,kw OR inventor*:ab,ti,kw | 8,160,215 |
| #1 | 'mental disease'/de OR 'mental stress'/exp OR 'mood disorder'/exp OR 'anxiety disorder'/exp OR 'Diagnostic and Statistical Manual of Mental Disorders'/exp OR 'anxiety'/de OR 'depression'/exp OR 'psychological distress':ab,ti,kw OR 'psychological stress':ab,ti,kw OR anxiety:ab,ti,kw OR anxieties:ab,ti,kw OR anxious:ab,ti,kw OR depress*:ab,ti,kw OR phobi*:ab,ti,kw OR panic:ab,ti,kw OR 'common mental disorder*':ab,ti,kw OR 'Acute Stress Disorder*':ab,ti,kw OR 'Post-Traumatic Stress Disorder*':ab,ti,kw OR 'Posttraumatic Stress Disorder*':ab,ti,kw OR PTSD:ab,ti,kw | 1,398,678 |

**EBSCO/PsycINFO Session Results (22 Jan 2021)**

| **#** | **Query** | **Results** |
| --- | --- | --- |
| S6 | S1 AND S2 AND S3 AND S4 AND S5 | 736 |
| S5 | TI ("Middle East*" OR Algeria* OR Bahrain* OR Chad* OR Comoros OR Djibouti* OR Egypt* OR Eritrea* OR Iraq* OR Israel* OR Jordan* OR Kuwait* OR Leban* OR Libya* OR Malta OR Maltes* OR Mauritania* OR Morocc* OR Oman* OR Palestin* OR Qatar* OR Saudi* OR Somalia* OR Sudan* OR Syria* OR Tanzan* OR Tunis* OR UAE OR Yemen* OR Cyprus OR Cypriot* OR Iran* OR Mali OR Niger OR Senegal* OR Turkey OR Turks OR arab OR arabic) OR AB ("Middle East*" OR Algeria* OR Bahrain* OR Chad* OR Comoros OR Djibouti* OR Egypt* OR Eritrea* OR Iraq* OR Israel* OR Jordan* OR Kuwait* OR Leban* OR Libya* OR Malta OR Maltes* OR Mauritania* OR Morocc* OR Oman* OR Palestin* OR Qatar* OR Saudi* OR Somalia* OR Sudan* OR Syria* OR Tanzan* OR Tunis* OR UAE OR Yemen* OR Cyprus OR Cypriot* OR Iran* OR Mali OR Niger OR Senegal* OR Turkey OR Turks OR arab OR arabic) OR KW ("Middle East*" OR Algeria* OR Bahrain* OR Chad* OR Comoros OR Djibouti* OR Egypt* OR Eritrea* OR Iraq* OR Israel* OR Jordan* OR Kuwait* OR Leban* OR Libya* OR Malta OR Maltes* OR Mauritania* OR Morocc* OR Oman* OR Palestin* OR Qatar* OR Saudi* OR Somalia* OR Sudan* OR Syria* OR Tanzan* OR Tunis* OR UAE OR Yemen* OR Cyprus OR Cypriot* OR Iran* OR Mali OR Niger OR Senegal* OR Turkey OR Turks OR arab OR arabic) | 68,984 |
| S4 | TI (Sensitivity OR Specificity OR ROC OR "Receiver Operating Characteristic*" OR "Receiver Operative Characteristic*" OR "Receiver Operating Curve*" OR "Receiver Operative Curve*" OR accuracy OR validit* OR validat* OR "Detection Limit*" OR "Limits of Detection" OR "Limit of Detection" OR "Predictive Value of Test*" OR "false positive" OR "false negative" OR likelyhood* OR likelihood* OR pretest OR "pre-test" OR posttest OR "post-test") OR AB (Sensitivity OR Specificity OR ROC OR "Receiver Operating Characteristic*" OR "Receiver Operative Characteristic*" OR "Receiver Operating Curve*" OR "Receiver Operative Curve*" OR accuracy OR validit* OR validat* OR "Detection Limit*" OR "Limits of Detection" OR "Limit of Detection" OR "Predictive Value of Test*" OR "false positive" OR "false negative" OR likelyhood* OR likelihood* OR pretest OR "pre-test" OR posttest OR "post-test") OR DE "Test Reliability" | 485,883 |
| S3 | DE "Psychodiagnostic Interview" OR TI ("reference standard*" OR "reference test*" OR "gold* standard*" OR clinical* OR clinician* OR diagnos* OR neuropsychiatric* OR "neuro-psychiatric*" OR interview* OR examination* OR schedule* OR structured OR "semi-structured" OR semistructured OR "symptom scale*" OR MINI OR CDI OR CIDI OR CIS OR SCID) OR AB ("reference standard*" OR "reference test*" OR "gold* standard*" OR clinical* OR clinician* OR diagnos* OR neuropsychiatric* OR "neuro-psychiatric*" OR interview* OR examination* OR schedule* OR structured OR "semi-structured" OR semistructured OR "symptom scale*" OR MINI OR CDI OR CIDI OR CIS OR SCID) OR KW ("reference standard*" OR "reference test*" OR "gold* standard*" OR clinical* OR clinician* OR diagnos* OR neuropsychiatric* OR "neuro-psychiatric*" OR interview* OR examination* OR schedule* OR structured OR "semi-structured" OR semistructured OR "symptom scale*" OR MINI OR CDI OR CIDI OR CIS OR SCID) | 1,234,025 |
| S2 | DE "Self-Evaluation" OR DE "Surveys" OR DE "Questionnaires" OR DE "Self-Report" OR DE "Rating Scales" OR DE "Testing" OR DE "Self-Disclosure" OR TI (questionnaire* OR "self-evaluation*" OR "self-report*" OR selfreport* OR "self-assessment*" OR "self-disclosure*" OR screening OR screener* OR index OR indices OR instrument OR instruments OR measure OR measures OR scale OR scales OR scaling OR survey OR surveys OR tool* OR test OR tests OR checklist* OR inventor*) OR AB (questionnaire* OR "self-evaluation*" OR "self-report*" OR selfreport* OR "self-assessment*" OR "self-disclosure*" OR screening OR screener* OR index OR indices OR instrument OR instruments OR measure OR measures OR scale OR scales OR scaling OR survey OR surveys OR tool* OR test OR tests OR checklist* OR inventor*) OR KW (questionnaire* OR "self-evaluation*" OR "self-report*" OR selfreport* OR "self-assessment*" OR "self-disclosure*" OR screening OR screener* OR index OR indices OR instrument OR instruments OR measure OR measures OR scale OR scales OR scaling OR survey OR surveys OR tool* OR test OR tests OR checklist* OR inventor*) | 1,763,624 |
| S1 | DE "Mental Disorders" OR DE "Psychological Stress" OR DE "Affective Disorders" OR DE "Major Depression" OR DE "Anxiety Disorders" OR DE "Acute Stress Disorder" OR DE "Post-Traumatic Stress" OR DE "Posttraumatic Stress Disorder" OR DE "Diagnostic and Statistical Manual" OR DE "Anxiety" OR DE "Depression (Emotion)" OR TI ("psychological distress" OR "psychological stress" OR anxiety OR anxieties OR anxious OR depress* OR phobi* OR panic "common mental disorder*" OR "Acute Stress Disorder*" OR "Post-Traumatic Stress Disorder*" OR "Posttraumatic Stress Disorder*" OR PTSD) OR AB ("psychological distress" OR "psychological stress" OR anxiety OR anxieties OR anxious OR depress* OR phobi* OR panic OR "common mental disorder*" OR "Acute Stress Disorder*" OR "Post-Traumatic Stress Disorder*" OR "Posttraumatic Stress Disorder*" OR PTSD) OR KW ("psychological distress" OR "psychological stress" OR anxiety OR anxieties OR anxious OR depress* OR phobi* OR panic OR "common mental disorder*" OR "Acute Stress Disorder*" OR "Post-Traumatic Stress Disorder*" OR "Posttraumatic Stress Disorder*" OR PTSD) | 621,220 |

**Scopus Session Results (22 Jan 2021)**

| **#** | **Query** | **Results** |
| --- | --- | --- |
| #6 | #1 AND #2 AND #3 AND #4 AND #5 | 2,117 |
| #5 | TITLE-ABS-KEY ("Middle East*" OR Algeria* OR Bahrain* OR Chad* OR Comoros OR Djibouti* OR Egypt* OR Eritrea* OR Iraq* OR Israel* OR Jordan* OR Kuwait* OR Leban* OR Libya* OR Malta OR Maltes* OR Mauritania* OR Morocc* OR Oman* OR Palestin* OR Qatar* OR Saudi* OR Somalia* OR Sudan* OR Syria* OR Tanzan* OR Tunis* OR UAE OR Yemen* OR Cyprus OR Cypriot* OR Iran* OR Mali OR Niger OR Senegal* OR Turkey OR Turks OR arab OR Arabic) | 1,102,964 |
| #4 | TITLE-ABS-KEY (Sensitivity OR Specificity OR ROC OR "Receiver Operating Characteristic*" OR "Receiver Operative Characteristic*" OR "Receiver Operating Curve*" OR "Receiver Operative Curve*" OR accuracy OR validit* OR validat* OR "Detection Limit*" OR {Limits of Detection} OR {Limit of Detection} OR "Predictive Value of Test*" OR {false positive} OR {false negative} OR likelyhood* OR likelihood* OR pretest OR {pre-test} OR posttest OR {post-test}) | 7,087,527 |
| #3 | TITLE-ABS-KEY ("reference standard*" OR "reference test*" OR (gold* PRE/1 standard*) OR clinical* OR clinician* OR diagnos* OR neuropsychiatric* OR "neuro-psychiatric*" OR interview* OR examination* OR schedule* OR structured OR {semi-structured} OR semistructured OR "symptom scale*" OR MINI OR CDI OR CIDI OR CIS OR SCID) | 14,763,517 |
| #2 | TITLE-ABS-KEY ("Self-Evaluation*" OR "self-report*" OR selfreport* OR "self-assessment*" OR "self-disclosure*" OR questionnaire* OR screening OR screener* OR index OR indices OR instrument OR instruments OR measure OR measures OR scale OR scales OR scaling OR survey OR surveys OR tool* OR test OR tests OR checklist* OR inventor*) | 18,301,133 |
| #1 | TITLE-ABS-KEY ({psychological distress} OR {psychological stress} OR anxiety OR anxieties OR anxious OR depress* OR phobi* OR panic OR "common mental disorder*" OR "Acute Stress Disorder*" OR "Post-Traumatic Stress Disorder*" OR "Posttraumatic Stress Disorder*" OR PTSD) | 1,221,552 |

**Wiley/Cochrane Library Session Results (22 Jan 2021)**

| **#** | **Query** | **Results** |
| --- | --- | --- |
| #6 | #1 AND #2 AND #3 AND #4 AND #5 | 271 |
| #5 | ((Middle NEXT East*) OR Algeria* OR Bahrain* OR Chad* OR Comoros OR Djibouti* OR Egypt* OR Eritrea* OR Iraq* OR Israel* OR Jordan* OR Kuwait* OR Leban* OR Libya* OR Malta OR Maltes* OR Mauritania* OR Morocc* OR Oman* OR Palestin* OR Qatar* OR Saudi* OR Somalia* OR Sudan* OR Syria* OR Tanzan* OR Tunis* OR UAE OR Yemen* OR Cyprus OR Cypriot* OR Iran* OR Mali OR Niger OR Senegal* OR Turkey OR Turks OR arab OR arabic):ab,ti,kw | 22,872 |
| #4 | (Sensitivity OR Specificity OR ROC OR (Receiver NEXT Operati* NEXT Characteristic*) OR (Receiver NEXT Operati* NEXT Curve*) OR accuracy OR validit* OR validat* OR (Detection NEXT Limit*) OR (Limit* NEXT of NEXT Detection) OR (Predictive NEXT Value NEXT of NEXT Test*) OR (false NEXT positive) OR (false NEXT negative) OR likelyhood* OR likelihood* OR pretest OR (pre NEXT test) OR posttest OR (post NEXT test)):ab,ti,kw | 145,235 |
| #3 | ((reference NEXT standard*) OR (reference NEXT test*) OR (gold* NEXT standard*) OR clinical* OR clinician* OR diagnos* OR neuropsychiatric* OR (neuro NEXT psychiatric*) OR interview* OR examination* OR schedule* OR structured OR (semi NEXT structured) OR semistructured OR (symptom NEXT scale*) OR MINI OR CDI OR CIDI OR CIS OR SCID):ab,ti,kw | 969,861 |
| #2 | ((Self NEXT Evaluation*) OR (self NEXT report*) OR selfreport* OR (self NEXT assessment*) OR (self NEXT disclosure*) OR questionnaire* OR screening OR screener* OR index OR indices OR instrument OR instruments OR measure OR measures OR scale OR scales OR scaling OR survey OR surveys OR tool* OR test OR tests OR checklist* OR inventor*):ab,ti,kw | 733,235 |
| #1 | ((psychological NEXT distress) OR (psychological NEXT stress) OR anxiety OR anxieties OR anxious OR depress* OR phobi* OR panic OR (common NEXT mental NEXT disorder*) OR (Acute NEXT Stress NEXT Disorder*) OR (Post-Traumatic NEXT Stress NEXT Disorder*) OR (Posttraumatic NEXT Stress NEXT Disorder*) OR PTSD):ab,ti,kw | 120,314 |
